# The integrated stress response promotes neural stem cell survival under conditions of mitochondrial dysfunction in neurodegeneration

**DOI:** 10.1111/acel.14165

**Published:** 2024-05-16

**Authors:** Mohamed Ariff Iqbal, Maria Bilen, Yubing Liu, Vanessa Jabre, Bensun C. Fong, Imane Chakroun, Smitha Paul, Jingwei Chen, Steven Wade, Michel Kanaan, Mary‐Ellen Harper, Mireille Khacho, Ruth S. Slack

**Affiliations:** ^1^ Department of Cellular and Molecular Medicine University of Ottawa Brain and Mind Research Institute University of Ottawa Ottawa Ontario Canada; ^2^ Department of Biochemistry, Microbiology and Immunology, Center for Neuromuscular Disease (CNMD), Ottawa Institute of Systems Biology (OISB), Faculty of Medicine University of Ottawa Ottawa Ontario Canada; ^3^ Department of Biochemistry, Microbiology and Immunology, Ottawa Institute of Systems Biology (OISB), Faculty of Medicine University of Ottawa Ottawa Ontario Canada

**Keywords:** adult neurogenesis, Hypoxia, intergrated stress response, metabolic adaptation, mitochondrial dynamics, neurodegeneration, Opa1

## Abstract

Impaired mitochondrial function is a hallmark of aging and a major contributor to neurodegenerative diseases. We have shown that disrupted mitochondrial dynamics typically found in aging alters the fate of neural stem cells (NSCs) leading to impairments in learning and memory. At present, little is known regarding the mechanisms by which neural stem and progenitor cells survive and adapt to mitochondrial dysfunction. Using Opa1‐inducible knockout as a model of aging and neurodegeneration, we identify a decline in neurogenesis due to impaired stem cell activation and progenitor proliferation, which can be rescued by the mitigation of oxidative stress through hypoxia. Through sc‐RNA‐seq, we identify the ATF4 pathway as a critical mechanism underlying cellular adaptation to metabolic stress. ATF4 knockdown in Opa1‐deficient NSCs accelerates cell death, while the increased expression of ATF4 enhances proliferation and survival. Using a Slc7a11 mutant, an ATF4 target, we show that ATF4‐mediated glutathione production plays a critical role in maintaining NSC survival and function under stress conditions. Together, we show that the activation of the integrated stress response (ISR) pathway enables NSCs to adapt to metabolic stress due to mitochondrial dysfunction and metabolic stress and may serve as a therapeutic target to enhance NSC survival and function in aging and neurodegeneration.

## Graphical abstract

Opa1 loss impairs mitochondrial function leading to early neurogenesis defects and cognitive decline. Abberant Mitochondrial fragmentation leads to oxidative stress and ATF4‐mediated integrated stress response. ATF4 regulates adaptive response through Slc7a11 resulting in neural stem cell survival.

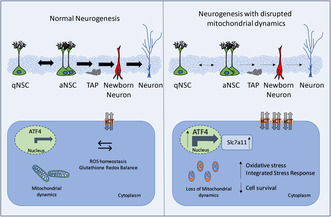

**Abbreviation**
Ascl1: Achaete‐scute homolog 1
ATF4: Activating transcription factor 4
ATP: Adenosine triphosphate
BF1: Brain factor‐1
DAPI: 4′,6‐diamidino‐2‐phenylindole
Dcx: Doublecortin
EdU: 5‐Ethynyl‐2'‐deoxyuridine
ETC: Electron transport chain
GSH: Glutathione
GSSG: Glutathione disulfide
IHC: Immunohistochemistry
ISR: Integrated stress response
NAD: Nicotinamide adenine dinucleotide
NADH: Reduced nicotinamide adenine dinucleotide
NSC: Neural stem cell
OCR: Oxygen consumption rate
OPA1: Optic atrophy protein 1
OXPHOS: Oxidative phosphorylation
PERK: Protein kinase R (PKR)‐like endoplasmic reticulum kinase
ROS: Reactive oxygen species
SGZ: Subgranular zone
SLC7A11: Solute Carrier Family 7 Member 11
SOX2: SRY‐box 2
SVZ: Subventricular zone
TAP: Transit amplifying progenitor
UMAP: Uniform Manifold Approximation and Projection
YFP: Yellow fluorescent protein

## 1 INTRODUCTION

Defective mitochondrial function has been attributed to aging and is often accelerated in neurodegenerative diseases. Mitochondrial disorders manifest through cumulative dysfunction in aging or through neonatal inheritance resulting in neurodegenerative and neurodevelopmental diseases. Mutations in the mitochondrial dynamics proteins, Drp1, Mfn1/2, and Opa1, have been shown to contribute to psychomotor, cognitive, and neurodegenerative disorders (Alexander et al., 2000; Delettre et al., 2000; Kijima et al., 2005; Longo et al., 2020; Züchner et al., 2004). We have recently shown that impaired mitochondrial dynamics alters the fate of neural stem cells (NSCs) leading to impairments in learning and memory (Khacho et al., 2016, 2017, 2019; Khacho & Slack, 2017). Thus, NSCs and their progeny are highly affected by mitochondrial dysfunction and metabolic stress in the developing, adult, and aging brain.

Continuous neurogenesis through adult NSC and progenitor cells plays an important role in learning and memory (Deng et al., 2010; Denoth‐Lippuner & Jessberger, 2021; Kee et al., 2007; Luna et al., 2019). The impairment of this process has been associated with neurodegenerative diseases in humans suggesting that a decline in adult‐born neurons contributes to the associated memory deficits (Moreno‐Jiménez et al., 2019; Terreros‐Roncal et al., 2021). In induced pluripotent stem cell (iPSC) models and murine neurogenesis, there is a clear distinction between the metabolism of undifferentiated NSCs and differentiated neurons. Quiescent NSCs and iPSC‐derived uncommitted NSCs are distinctly glycolytic; however, upon differentiation, metabolic regulators such as PGC1a and TFAM trigger mitochondrial biogenesis and metabolic shifts (Beckervordersandforth et al., 2017; Inak et al., 2021; Zheng et al., 2016). Furthermore, studies from our laboratory and others have shown that the maintenance of mitochondrial dynamics is essential for ongoing neurogenesis (Iwata et al., 2020; Khacho et al., 2016). Specifically, mitochondrial shape is regulated in a stage‐specific manner during NSC lineage progression in development and in the adult niche (Beckervordersandforth et al., 2017; Iwata et al., 2020; Khacho et al., 2016). Importantly, changes in mitochondrial dynamics have a direct influence on mitochondrial metabolism and signaling, which subsequently alter cell fate decisions through gene regulation (Beckervordersandforth et al., 2017; Inak et al., 2021; Iwata et al., 2020; Khacho et al., 2016, 2017; Zhang et al., 2016).

Despite the evidence linking mitochondrial function to NSC fate decision and learning and memory, little is known as to how NSCs and their progeny respond and adapt to mitochondrial impairments typically seen in aging and neurodegeneration. Since Opa1 plays a critical role in the regulation of mitochondrial structure and function and is implicated in a number of neurodegenerative diseases (Alavi & Fuhrmann, 2013; Frezza et al., 2006; Patten et al., 2014), we generated an Opa1‐inducible knockout in adult NSCs as a model to study the effects of mitochondrial dysfunction on adult neurogenesis (Kane et al., 2017; Ramonet et al., 2013). We report in this study that loss of Opa1 and mitochondrial dysfunction leads to a decline in neurogenesis due to impaired stem cell activation and proliferation. Importantly, we identify ATF4 as a critical player in maintaining NSC survival during these conditions by enabling cellular adaptation to stress. Furthermore, ATF4 facilitates glutathione production as a regulatory target to sustain stem cell function and survival under conditions of mitochondrial dysfunction typical of aging and neurodegeneration.

## 2 METHODS

### 2.1 Animals

All the animal protocols were approved by the Animal Care Ethics Committee of the University of Ottawa and adhered to the Guidelines of the Canadian Council on Animal Care. Animals were genotyped according to published protocols: primers are listed in Table S2.

We developed a tamoxifen (TAM)‐inducible reporter mouse Nestin‐CreER^T2^ (Cicero et al., 2009); R26‐stop‐enhanced yellow fluorescent protein (YFP; Srinivas et al., 2001) mice, a gift from S. Baker. Control transgenic animals (CT) were made by crossing Nestin‐CreER^T2^ and R26‐stop‐enhanced YFP (Nestin‐CreER^T2^:Rosa26YFP) mice. To generate Opa1‐inducible knockouts (Opa1 cKO) in nestin‐expressing cells, Opa1*flox*/*flox* mice, a kind gift from Dr. Hiromi Sesaki, were crossed with Nestin‐CreER^T2^:Rosa26YFP to obtain Nestin‐CreER^T2^:Rosa26YFPflox:Opa1flox line (Z. Zhang et al., 2011). All the mice were maintained on a C57/BL/6 background. All Nestin‐CreER^T2^ animals used were heterozygotes for Cre expression. Both females and males were used in all experiments, and all animals were 6–10 weeks old upon initial treatment.

BF1^Cre^:Opa1 knockouts (Hebert & McConnell, 2000) were generated by crossing BF1^Cre/+^:Opa1^flox/+^ with Opa1^flox/flox^ mice. The BF1^+/+^ littermates were used as CT and BF1^Cre/+^:Opa1^flox/flox^ were used as Opa1 knockouts. Subtle gray mice C3H/HeSnJ‐Slc7a11sut/J (Sut mice) JAX Strain #:001310 (Sato et al., 1999); and background control C3H/HeSnJ (wild type, WT) JAX Strain #:000661 were maintained as individual lines and used at mentioned time points for histology. Both females and males were used in all experiments, and all animals were 5–8 weeks old upon initial isolation for neurosphere culture.

### 2.2 Tamoxifen injection

To generate the inducible Opa1 knockout in adult NestinCreER^T2^ animals, CT and Opa1cKO mice were administered TAM at 200 mg/kg/d for 5d (oral gavage; dissolved in 10% ethanol/90% corn oil) (Fong et al., 2022). Animal weight at the beginning was used for calculating TAM dosage; weight after final injection was monitored and wellness checks were performed as per the University of Ottawa standards until euthanization.

### 2.3 Contextual fear conditioning analysis

Fear conditioning experiments were performed using the PhenoTyper box and Ethovision 10 XT video tracking system (Noldus Information Technology, North America) as previously described (Pham et al., 2009) with minor modifications in order to assess fear memory formation (Maren et al., 2013; Saxe et al., 2006). The method used for the fear conditioning test, including the use of the foot shock sensitivity test to determine optimal shock, was similar to our previous publication (Khacho et al., 2016). The protocol used for contextual fear conditioning was similar to Cancino et al. (2013). Briefly, on Days 1 and 2, mice were handled in the testing room for habituation, and on Day 3, mice were pre‐exposed to the conditioning chamber for 10 min in the absence of a shock. On Day 4, approximately 24 h after pre‐exposure, mice were placed in the same conditioning context and 5 s later received an immediate foot shock (2 sec, 0.7 mA) and were removed from the context after 1 min. Twenty‐four hours and 7 days after shock training, mice were placed into the same conditioning context and freezing was assessed for a 6‐min period.

### 2.4 Histology

Systemic tissue perfusion and fixation were performed as previously described (McClellan et al., 2007). Briefly, mice were flushed with 0.9% saline and then perfused transcardially with 4% paraformaldehyde (PFA), brains were immersed in 4% PFA overnight, and then transferred to 20% sucrose in PBS for long‐term storage. After removing the cerebellum to establish a flat coronal edge, fixed brains were frozen between −30 and − 40°C in isopentane prior to cryosectioning. Brains were serially sectioned into 30 μm coronal sections for neurogenesis markers and 14 μm coronal sections for mitochondrial staining, maintaining the temperature between −20°C and −25°C and collecting the subventricular zone (SVZ) and the subgranular zone (SGZ) of the dentate gyrus of the hippocampal structure. Wells containing free‐floating 1:9 serial sections were stored long‐term in PBS at 4°C. Brains sectioned for mitochondrial staining were slide‐mounted and stored long‐term at 4°C.

### 2.5 EdU incorporation

In vivo labeling of proliferating cells was achieved by giving an intraperitoneal injection of 50 μg of EdU (5‐ethynyl‐2′‐deoxyuridine) per gram of body weight (Clickbase, BCK647‐IV‐IM‐M) as previously described (Furutachi et al., 2015). In brief, mice were injected with one dose of EdU daily for 7 days prior to the TAM injection and loss of Opa1, in order to allow labeling proliferating NSCs. Following TAM injection, SGZ cells were allowed to divide for 4 weeks before harvest.

### 2.6 Immunohistochemistry and imaging, cell quantification, and statistical analysis

Immunohistochemistry was performed as previously described (Vandenbosch et al., 2016). Z‐stacks spanning 10 μm of post‐processing section width, with 2.0 μm spacing between Z‐positions were used for imaging. Single labeled sections were quantified on an Olympus BX‐51 microscope. Immunohistochemistry to assess mitochondrial length was performed as previously described (Royea & Khacho, 2022; Triolo et al., 2023). Antibodies are listed in Table S1. Sections for quantification of colocalized cells were imaged using LSM880 Airyscan Confocal Microscope (Zeiss), employing a 40× objective or a drop of immersion oil onto a 63× oil and spacing of 0.16 μm between Z‐positions, as required. Images were analyzed using Fiji software (Schindelin et al., 2012).

All statistical comparisons in this study were performed using an unpaired two‐tailed *t* test. Differences were considered significant with a *p*‐value of <0.05 (*), **p < 0.01, ***p < 0.001, ****p < 0.0001. Unless otherwise stated, all data are presented as the arithmetic mean, plus or minus the standard deviation (mean ± SD). For each experiment, biological replicates of 3–6 animals per genotype were used.

### 2.7 Fluorescence‐activated cell sorting (FACS) and single‐cell RNA sequencing (sc‐RNA‐seq)

Hippocampal YFP+ cells were isolated as described in (Iqbal et al., 2022). YFP+ cells were collected from three mice of each genotype after viability screening with propidium iodide staining (ThermoFisher, Cat. # P3566) on a Beckman Coulter MoFlo platform in the Flow Cytometry and Cell Sorting Facility located in the Ottawa Hospital Sprott Center (Ottawa, Canada). Freshly collected YFP‐positive cells were sent to StemCore Laboratories located in the Ottawa Hospital Sprott Center, for both CT and Opa1 cKO samples. The transcriptome of viable single‐cells was analyzed individually for each genotype using the 10× Genomics Chromium single‐cell Assay platform (Zheng et al., 2017). A single‐cell RNA library was created following the single‐cell 3' Reagent Kits v2 user guide (Cat: CG0052, 10× Genomics) on the Chromium Single‐cell Instrument (10× Genomics) using Single‐cell 3' Library & Gel Bead Kit v2 and Chip Kit (P/N 120236 and P/N 120237, 10× Genomics). The cDNA library was then purified with SPRIselect (Beckman Coulter). Agilent Fragment Analyzer was used to evaluate the size distribution and yield (Agilent). Single‐cell RNA sequencing (sc‐RNA‐seq) was performed on Illumina NextSeq 500 instrument (Illumina) using the following parameters 28 bp Read1, 8 bp I7 Index, 0 bp I5 Index, and 98 bp Read2. Single‐end reads were aligned to the reference genome, mm10, using the CellRanger pipeline (10× Genomics), after demultiplexing cell barcodes and UMI (unique molecular identifiers) barcodes. The output cell‐gene matrix contains UMI counts by genes and by cell barcodes. Gene expression profiles were then mapped using Seurat v3 (or latest) and unsupervised clustering through nonlinear Uniform Manifold Approximation and Projection (UMAP) in R studio (Stuart et al., 2019).

### 2.8 sc‐RNA‐seq data analysis

Cells were filtered with the following criteria for further analysis: (1) cells with more than 600 genes recorded; (2) with less than 7500 genes recorded; (3) with mitochondria content less than 20% of total RNA counts; and (4) with detectable YFP expression. RNA reads were normalized with the function of sctransform “SCT”. Based on normalized RNA reads, cells were clustered using the following parameters: (1) dims =1:8; and (2) resolution = 0.2. Nine cell clusters were identified based on normalized expression profiles of each cell. Based on the original RNA reads, cluster‐specific markers were calculated with the function of FindAllMarkers. Differential expression between CT and Opa1 cKO for each cluster was analyzed with the function of FindMarkers. Heatmaps of cluster‐specific genes were mapped on normalized data. FeaturePlot (UMAP) and VlnPlot (violin plot) of specific genes were mapped on original RNA reads.

Cell clusters with characteristics of the neurogenic lineage were sub‐grouped to show differential expression of selected genes. Activated NSC, transit amplifying progenitors (TAPs), and newborn neurons were sub‐grouped and reclustered using the following parameters: (1) dims =1:12; and (2) resolution = 0.1. Gene expression and RNA velocity data were generated in Seurat based on normalized data and analyzed in Python‐based Spyder program using the “deterministic” mode (La Manno et al., 2018; Stuart et al., 2019). RNA Velocity is used to predict cell fate based on mRNA post‐transcriptional processing (Bergen et al., 2021; La Manno et al., 2018).

### 2.9 FACS and RNA isolation

The isolation of YFP+ cell population from SVZ or SGZ were performed as previously described (Iqbal et al., 2022), employing tissue micro‐dissected from mice 30 days post‐TAM injection. The YFP+ cell population was FACS‐isolated using a cutoff of 488‐FL‐Log‐Height ≥ 102. For RNA‐Seq input, cell pellets containing minimum 200,000 YFP+ cells were frozen, and RNA isolated using the Arcturus® PicoPure® RNA Isolation Kit (ThermoFisher). Isolated RNA was used for quantitative RT‐PCR validation of knockout or differential gene expression.

### 2.10 MitoSox flow cytometry assay

Embryonic and SGZ NSCs were allowed to grow in culture and form neurospheres for 5 and 10 days respectively. They were then collected and dissociated into single‐cell suspension. Cells were incubated with MitoSOX Red for reactive oxygen species (ROS) measurement at a concentration of 1 or 5 μM (Molecular Probe) for 15–20 min followed by one wash with PBS before fluorescence measurements, as previously described (Khacho et al., 2016). FACSCalibur (BD), FlowJo, and Floreada software were used for analysis.

### 2.11 Quantitative RT‐PCR

Samples for RNA quantification were prepared using the Rotor‐Gene SYBR® Green PCR Kit (Qiagen) and quantified on a Qiagen Rotor‐Gene Q Real‐Time PCR Machine. Primers used are listed in Table S2, and RNA quantifications are expressed relative to β‐actin (normalized to 1.0).

### 2.12 Plasmids and Lentiviral vector production

Lentivirus vectors shRNA scramble control (shCtrl sequence; 5′‐CAACAAGATGAAGAGCACCAA‐3′) and mouse‐specific shRNA knockdown to OPA1 (shOPA1 sequence; 5′‐GCCTGACTTTATATGGGAAAT‐3′) were prepared using previously established protocols (Khacho et al., 2016). For ATF4 manipulation, pLVX‐scramble‐mCherry lentiviral expression vector construct was commercially obtained (Clontech 631,987) and used as control. shATF4 knockdown (5′‐CCAGAGCATTCCTTTAGTTTA‐3′) to generate pLV‐U6‐[shAtf4]‐mCherry were ordered from Vectorbuilder. ATF4 overexpression from Origene (MR205957) was cloned into the expression vector to generate pLVX‐E2F1a‐Atf4‐Myc‐DDK‐V5‐IRES‐mCherry. Lentiviruses were produced in 293 T HEK cells using polyethylenimine (PEI) transfection, and purified by ultracentrifugation, following established protocols (Tang et al., 2015), and was resuspended in PBS in small aliquots. Viruses with reporter were live titered using 293 T cells. QuickTiter™ Lentivirus Titer Kit (VPK‐107) was used to titer shCtrl and shOpa1 viruses using p24 antigen. For use in vitro, cells from embryonic cortex, SGZ and SVZ NSCs were transduced with lentiviral particles at a multiplicity of infection (MOI) of 15, before neurosphere differentiation after 5–7 days.

### 2.13 In vitro neurosphere and monolayer culture

Embryonic cortices were collected at embryonic day E12‐13 as previously described (Khacho et al., 2016). After removing meninges, the cortices were passed through a 1.0 mL pipette tip. The cell suspension was then filtered through a 70 μm mesh filter. The single‐cell suspension obtained was treated with appropriate lentivirus for 2 h at 37°C prior to seeding. DMEM/F12 (Gibco 11,330–032) supplemented with B27 without vitamin A (Gibco 12,587,010), EGF (Sigma E‐1257), bFGF (Sigma F‐0291), heparin (Sigma H‐3149), and penicillin/streptomycin (Gibco 1570–063) to encourage neurosphere formation. SVZ and SGZ regions were dissected in ice‐cold artificial cerebrospinal fluid (ACSF) solution and dissociated following a previous protocol (Iqbal et al., 2022). Briefly, tissues collected were digested in papain solution for 10 min at 37°C and cells were passed through a 22% (v/v) PBS‐buffered Percoll solution. Cells were plated at a concentration of 50 cells/μL and 10 cells/μL for SGZ and SVZ, respectively. Neurospheres were collected after 5–10 days in culture. For hypoxia experiments, cells were placed in Biospherix X3 Xvivo System and incubated in 1% O_2_ levels; 5% CO_2_ and 80% humidity.

### 2.14 ATP assay

Cells from the developing cortex of CT and Opa1 cKO were incubated in a black wall optical bottom 96‐well plate with or without 10 μM oligomycin. ATP concentrations were measured with the CellTiter‐Glo Luminescent Assay (Promega) using a BioTek Synergy H1 microplate reader according to the manufacturer's protocol. ATP concentrations were normalized to cell counts determined by trypan blue dye exclusion. Data were collected from multiple replicate wells for each experiment.

### 2.15 Nicotinamide adenine dinucleotide (NAD+) measurement

The levels of NAD^+^ and its reduced form NADH were measured using the NAD^+^ /NADH Quantitation Colorimetric Kit (BioVision K337‐100). Neurospheres in different conditions were lysed using the lysis buffer provided by the supplier. Colorimetric reading was detected using BioTek Synergy H1 microplate reader.

### 2.16 Cellular oxygen consumption

The Seahorse XF24 Extracellular Flux Analyzer (Seahorse Biosciences) was used to measure oxygen consumption in cells. Cells derived from neurosphere cultures were seeded onto the Cell‐Tak (22.4 μg/mL)‐coated 24‐well Seahorse plates at a density of 200,000 cells/well in 100 μL stem cell media (SCM) supplemented with additional 0.5 mM sodium pyruvate. Plates were immediately spun at 200 × g for 1 min and allowed to stop without brakes and then placed at 37°C for 25 min. 500 μL SCM was slowly added to each well, followed by an additional incubation of 20 min at 37°C prior to loading into the XF Analyzer. Following measurements of resting respiration, cells were treated sequentially with oligomycin (1 μg/μL) to measure the non‐phosphorylating oxygen consumption rate (OCR), carbonyl cyanide‐p‐trifluoromethoxyphenylhydrazone (FCCP) (2 μM) to get the maximal OCR, and antimycin A (1 μM) to measure the extramitochondrial OCR. Each measurement was taken over a 2‐min interval followed by 2 min of mixing and 2 min of incubation. Three measurements were taken for the resting OCR: after oligomycin treatment, after FCCP, and after antimycin A treatment.

### 2.17 Western blot

Cell pellets of cultured neurospheres were washed with PBS and lysed with 4% SDS in PBS, samples were sheared by passing through a 26‐gauge needle to denature DNA. Lysates were spun at high speed to remove particles. Primary antibodies were incubated overnight at 4°C. A secondary antibody conjugated to horseradish peroxidase (Jackson ImmunoResearch) was used and detected by Clarity™ Western ECL Substrate (Bio‐Rad). Chemiluminescence signal was captured in either radiograph films or Bio‐Rad ChemiDoc imaging system. Western blot bands were quantified using ImageJ (Schindelin et al., 2012).

### 2.18 In vitro cell staining

Dissociated single cells with appropriate conditions were grown in 8‐well multichamber slides (Thermo Fisher Scientific 154941PK). At the endpoint, cells were washed with cold PBS and fixed with 4% PFA for 20 min, washed 3 × 10 min with PBS, and blocked in 5% bovine serum albumin (BSA) in PBS followed by overnight incubation in primary antibody at 4°C. A secondary fluorophore antibody and DAPI to visualize nuclear DNA were added and cells were imaged using an Observer D1 fluorescent microscope.

### 2.19 Chromatin immunoprecipitation (ChIP)

ChIP assays followed by quantitative real‐time PCR (RT‐qPCR) were performed as previously described (Fong et al., 2022). 10 μg of cross‐linked chromatin was incubated with 0.8 μg antibodies in ChIP assays.

### 2.20 Chromatin immunoprecipitation‐sequencing (ChIP‐seq) analysis

ChIP‐seq data on ATF4 in mouse embryonic fibroblasts (MEFs) was performed as described previously (Han et al., 2013) and obtained from NCBI as Short Read Archive (SRA) under accession number SRP010861. Sequencing read data were analyzed following ENCODE pipeline. Technical or biological replicates were combined into single FastQ files and aligned to GRCm38 (mm10) version of the mouse genome.

### 2.21 Glutathione (GSH) and glutathione disulfide (GSSG) measurement by high‐performance liquid chromatography

GSH and GSSG levels were determined by Agilent 1100 series HPLC system. Briefly, the neurospheres were cultured in 100 mm petri dishes under the indicated conditions. At the endpoint, neurospheres were collected and washed with ice‐cold PBS, and lysed on ice for 20 min in 1:1 homogenization buffer made of 125 mM sucrose, 1.5 mM EDTA, 5 mM Tris, 0.5% trifluoroacetic acid (TFA), and 0.5% mycophenolic acid (MPA) in 50% mobile phase (10% HPLC grade methanol, 0.09% TFA—0.2 μm filtered). Homogenates were spun at 14,000 x *g* for 20 min at 4°C. Supernatant of each sample was collected and processed in duplicate using an Agilent HPLC system equipped with a Pursuit C18 column (150 × 4.6 mm, 5 μm; Agilent Technologies) operating at a flow rate of 1 mL/min and detected at 215 nm. Standards of GSH and GSSG were made in the same 1:1 homogenization buffer at the indicated concentrations. All values were normalized to the amount of protein in each sample.

## 3 RESULTS

### 3.1 Neurogenesis is impaired after Opa1 knockout in the adult hippocampus

**FIGURE 1:**
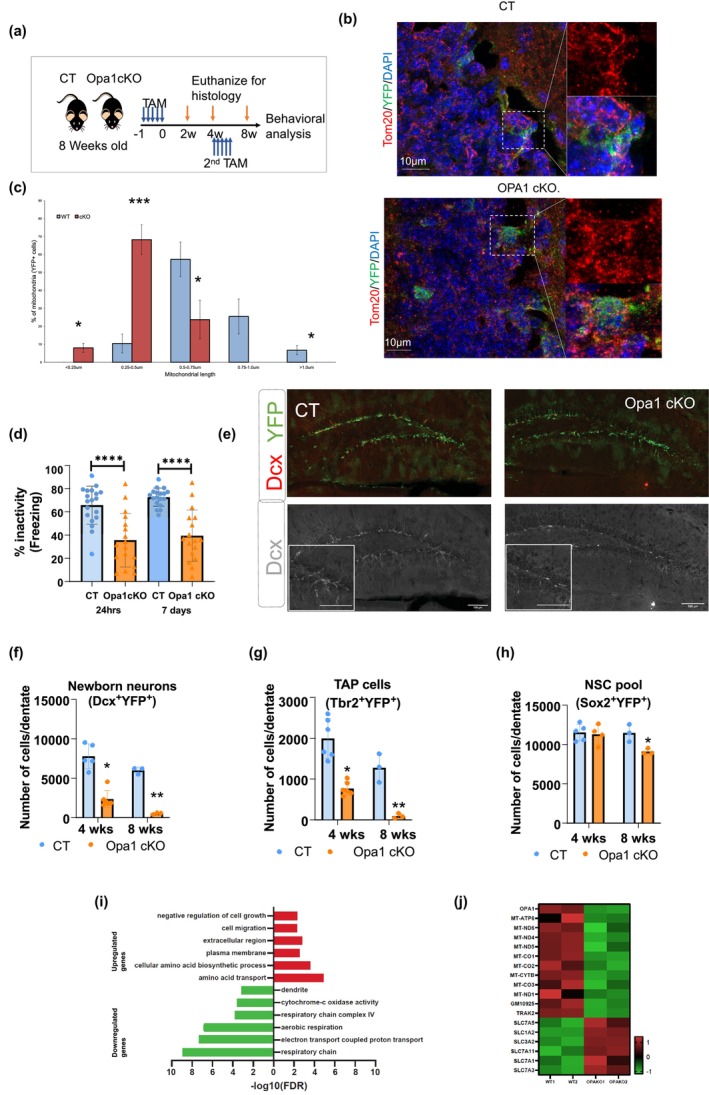
Opa1 loss impacts NSC commitment and neurogenesis in adult dentate gyrus. (a) Experimental setup. (b) Representative confocal images of coronal sections showing mitochondrial morphology (Tom20 in red), Nestin/YFP+ cells in green, and DAPI in blue from control transgenic (CT) and Opa1 cKO animals, 4 weeks post tamoxifen administration. (Scale bar = 10 μm). (c) Mitochondrial length in YFP+ cells in control transgenic (CT) and Opa1 cKO animals. *n* ≥ 40 mitochondria per condition, mean ± standard error of mean (SEM). (d) The graph indicates the percentage immobility in the same context at 24 h and 1 week following exposure to shock in Opa1 CT and Opa1 cKO animals (CT, *n* = 16; Opa1 cKO, *n* = 17 animals). Data are presented as mean ± SD (*p < 0.05, **p < 0.01, and ***p < 0.001, Student's *t* test). (e) Representative images of coronal sections of the dentate gyrus were immunostained for Dcx (red), YFP (green), and nuclear stain DAPI (blue) from control transgenic (CT) and Opa1 cKO animals 4 weeks post‐TAM administration and insets of magnified fluorescent images of Dcx (Scale bar = 100 μm) (f–h) Bar graphs representing the total cell number per dentate of Dcx (Newborn neurons), Tbr2 (Transit Amplifying Progenitors, TAP), and Sox2 (stem cell pool)‐expressing cells that are colocalized with YFP^+^ cells at the listed time in the listed genotype (*n* = 3–6 animals); Data are presented as mean ± SD (*p < 0.05, **p < 0.01, and ***p < 0.001, Student's *t* test). (i) GO term analysis of top upregulated genes (shown in red) and downregulated genes (shown in green) in RNA‐seq of SVZ sorted YFP+ cell of wild type (WT) versus Opa1 cKO (KO) animals. (j) Heatmap of the significantly upregulated and downregulated genes related to mitochondrialfunction (relative gene expression range from green −1 to red 1).

To generate a model of mitochondrial/metabolic stress in adult NSCs and their progeny, we used a TAM‐inducible conditional knockout of Opa1 (Opa1cKO) in adult NSCs (Figure 1a; Figure S1A). In this model, loss of Opa1 caused severe mitochondrial fragmentation in the population of YFP+ cells of the adult SGZ (Figure 1b,c). To elucidate the impact of mitochondrial dysfunction on adult hippocampal neurogenesis, we performed a histological analysis in the adult hippocampus of Opa1cKO and CT at 2, 4, and 8 weeks post TAM treatment, and behavioral assessments at the 8‐week time point (Figure 1a). We then examined cellular changes related to neurogenesis in the SGZ at 2, 4, and 8 weeks post TAM injection. While no detectable differences were observed at 2 weeks post TAM injection (Figure S1B), there was a drastic decrease in the generation of Dcx‐expressing newborn neurons at 4 (70% reduction) and 8 (90% reduction) weeks (Figure 1e,f) subsequently leading to a significant decrease in mature NeuN‐expressing neurons at 8 weeks post TAM (58.74% reduction) (Figure S1E,F). Opa1cKO also resulted in a 61% and 97% decline in the number of Tbr2^+^YFP^+^ expressing TAPs, 4 and 8 weeks post‐TAM respectively (Figure 1g; Figure S1C). To assess memory formation in Opa1 knockout, we performed contextual fear conditioning. Here, CT mice showed high pre‐shock activity and exhibited a significant “freezing” behavior at 24 h and 7 days post‐shock, whereas Opa1cKO mice exhibited a significant reduction in “freezing” behavior (Figure 1d), indicating an impairment in contextual memory. These results support our hypothesis that disruption in mitochondrial dynamics leads to impaired neurogenesis and deficits in learning and memory. To determine if the underlying memory deficit originates from a diminishing pool of stem/progenitor cells, we quantified the total number of adult neural stem/progenitor cells co‐expressing Sox2/YFP. While no changes were detected at 2 and 4 weeks (Figure 1h; Figure S1B,C), there was a slight but significant 20% decline in total Sox2^+^ cells at 8‐weeks post‐TAM (Figure 1h). In addition, RNA‐seq analysis of Opa1‐deficient FACS‐sorted cells from the adult SVZ showed a global decrease in key oxidative phosphorylation (OXPHOS) and mitochondrial gene expression consistent with impaired mitochondrial function (Figure 1i,j; Figure S1D), validating that mitochondria are disrupted within adult NSCs in this model. These data suggest that the decline in neurogenesis observed in this model may originate from mitochondrial defects within the NSC pool.

### 3.2 Loss of OPA1 and mitochondrial disruption causes defects in NSC activation and proliferation

**FIGURE 2:**
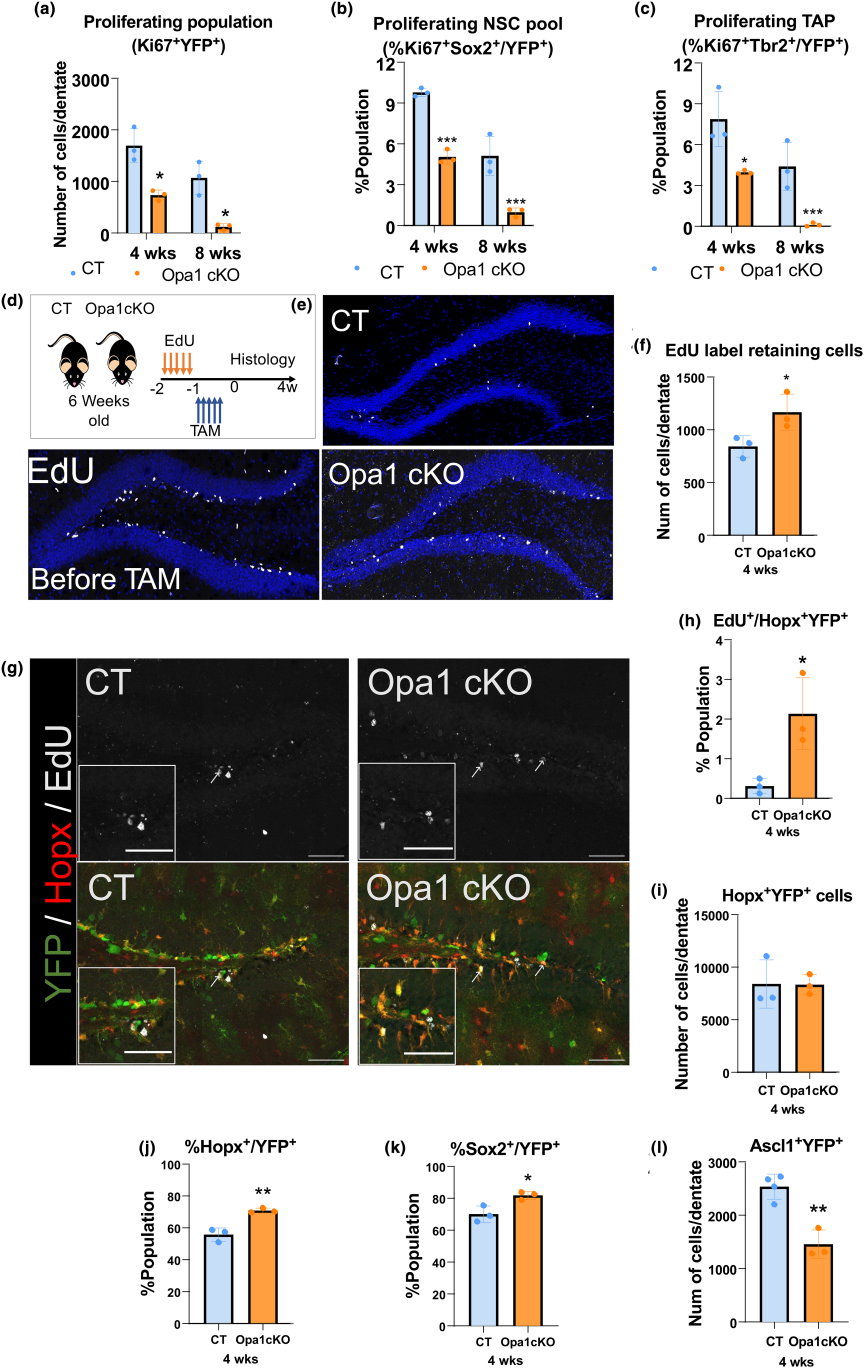
Opa1 loss impacts NSC proliferation and activation. (a) Bar graphs representing the total cell number per dentate of Ki67+ (proliferating cells). (b) Percentage of proliferating NSCs (Ki67^+^Sox2^+^ of total YFP^+^ cell in adult dentate at the listed time in the listed genotype. (c) Percentage of proliferating TAP cells (Ki67^+^Tbr2^+^ of total YFP^+^ cell in adult dentate. (d) Experimental setup. (e) Representative images of the adult dentate gyrus labeled with EdU at the given time for the listed genotype. (f) Total EdU+ cells are quantified to show the number of EdU retaining cells at 4 weeks post‐TAM in the dentate gyrus. (g) Representative images of Hopx^+^ cells retaining EdU label at 4 weeks post‐TAM in CT and Opa1 cKO sections (Marked by the arrow) and insets of magnified fields for the different markers. Scale bar = 100 μm. (h) Percentage of EdU^+^Hopx^+^YFP^+^ of total Hopx^+^YFP^+^ cells at 4 weeks post‐TAM in adult dentate. (i) Bar graph of the total number of Hopx^+^YFP^+^ cells retained at 4 weeks post‐TAM. (j) Percentage of Hopx^+^ cells of total YPF^+^ cells at 4 weeks post‐TAM represented in bar graphs. (k) Percentage of Sox2^+^ cells of total YPF^+^ cells at 4 weeks post‐TAM represented in bar graphs. (l) Bar graph of the total number of Ascl1^+^ cells (activation marker) at 4 weeks post‐TAM per dentate that are colocalized with YFP^+^ cells. (*n* = 3–4 animals); Data are presented as mean ± SD (*p < 0.05, **p < 0.01, and ***p < 0.001, Student's *t* test).

In order to understand the underlying cause for a decline in the number of NSCs, we measured NSC proliferation. We first observed a general decrease in global proliferation of Opa1cKO NSCs (Ki67^+^ staining) in total YFP^+^ cells (Figure 2a). Furthermore, although most adult NSC/progenitor cells survived and were maintained at 4 weeks, there was a significant decrease in proliferating NSC/progenitor cells (Ki67^+^Sox2^+^/YFP^+^) at 4 (60%) and 8 weeks (95%) post‐TAM (Figure 2b), as well as proliferating TAP population (Ki67^+^Tbr2^+^/YFP^+^) at 4 (50%) and 8 weeks (95%) post‐TAM (Figure 2c). These results reveal a significant proliferation defect in adult NSC/progenitor cells in Opa1cKO brains that may account for the severe deficit in the generation of newborn neurons in the adult SGZ.

To determine whether the quiescent NSCs also exhibit a defect in activation and cell cycle re‐entry, we performed an EdU retention assay (Figure 2d). We reasoned that the EdU label would be diluted in actively dividing cells, such as activated NSCs and neural progenitors, but would be retained in quiescent/slowly dividing cells. In the Opa1cKO mice, more cells in the SGZ retained EdU labeling compared to CT mice (Figure 2e,f). Interestingly, cells retaining EdU in Opa1 cKO mice did not co‐express Ki67, suggesting that they do not exist within the cell cycle and may represent the quiescent population of NSCs (Figure S2A–D). To confirm that the EdU retaining cells were NSCs, we used Hopx, a marker for NSC in the SGZ (Berg et al., 2019) to colabel with EdU^+^ cells in the dentate gyrus (Figure 2g,h). As seen with Sox2^+^ cells at 4 weeks post‐TAM (Figure 1f), the total Hopx^+^ cells among the YFP^+^ population were similar between CT and Opa1 cKO dentate gyrus (Figure 2i); however, there was a significant (7‐fold) increase in EdU retention in Hopx^+^YFP^+^ cells in Opa1cKO relative to littermate CT mice (Figure 2h). Thus, the increased number of EdU‐retaining Hopx^+^ cells in Opa1cKO suggests an impairment in adult NSC activation. A concomitant increase in the percentage of Sox2^+^ and Hopx^+^ quiescent NSC pool revealed an impairment in NSC proceeding to activation (Figure 2j,k). This is further confirmed by a 43% decrease in activated NSCs Ascl1^+^YFP^+^ cells in Opa1 cKO dentate gyrus at 4‐weeks post‐TAM (Castro et al., 2011; Raposo et al., 2015) (Figure 2l). Taken together, our results reveal a significant impairment in NSC activation, demonstrated by an increase in the number of long‐term EdU‐retaining Hopx cells, a decrease in NSC proliferation, and a decrease in the number of activated Ascl1‐expressing NSC/progenitor cells.

### 3.3 Mitochondrial dysregulation signals a stress response by early activation of the ATF4 pathway in the NSC pool

**FIGURE 3:**
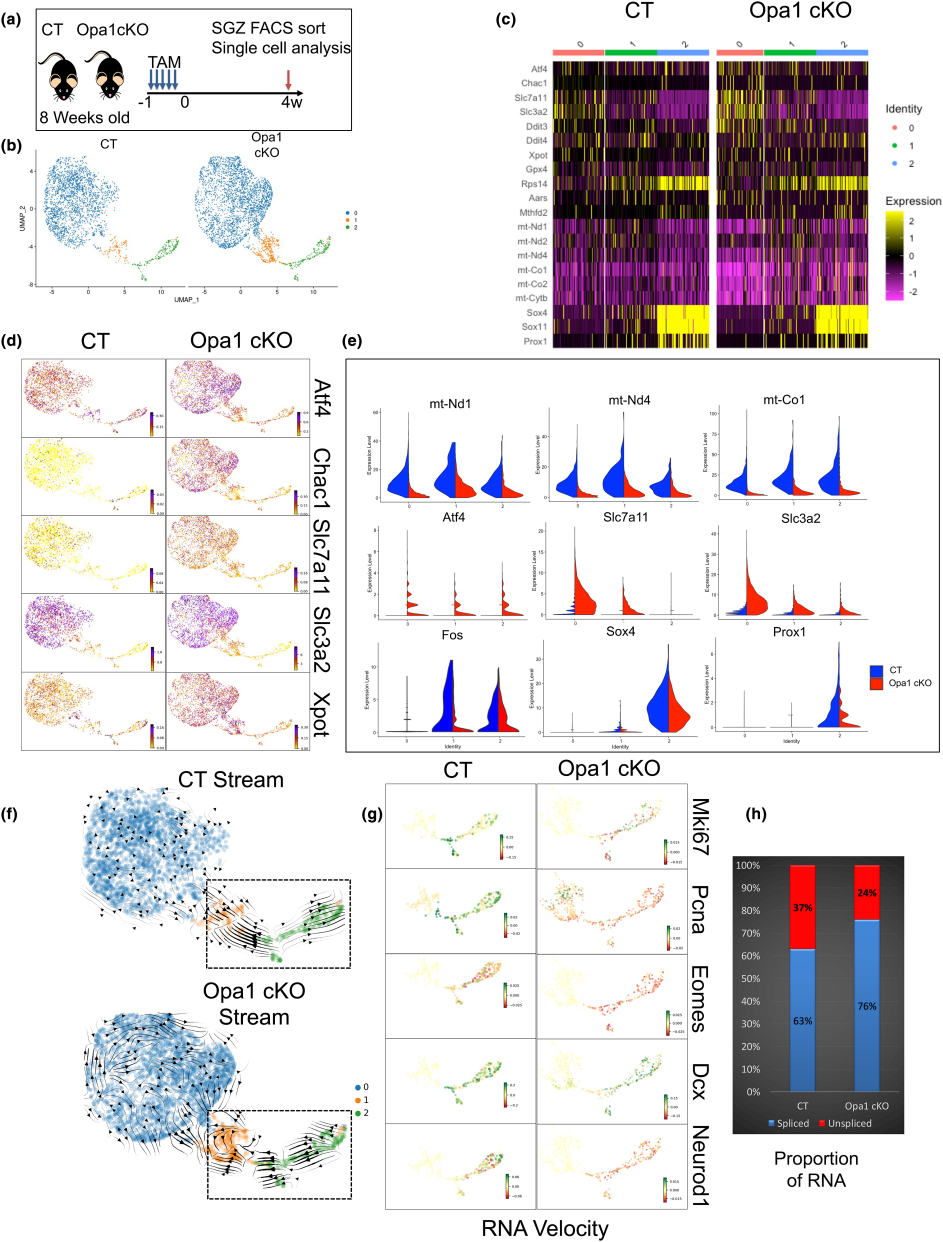
Single‐cell RNA‐Seq analysis identifies Atf4 stress response pathway downstream of mitochondrial dysfunction. (a) Experimental setup. (b) UMAP scatterplot showing the distribution of CT and Opa1 cKO‐derived cells clusters. (c) Heatmap of cluster‐specific and genotype‐specific differential gene expression. (d) UMAP of individual genes that are differentially regulated in all the clusters highlighting Atf4 pathway. (e) Violin plots representing mitochondrial gene expression, stress response genes, and differentiation genes in each cluster split by sample. (f) RNA velocity analysis shows the differentiation direction shown by the vectors separated by sample. (g) Panels of magnified views of the transition between cluster 1 (activated NSCs) and cluster 2 (Differentiating NSCs). (h) The proportion of spliced and unspliced RNA in all the clusters split by sample.

To better understand the impact of mitochondrial dysfunction on the different cell populations of the neuronal lineage and to identify downstream mechanisms linked to neurogenesis defects and altered memory formation, we performed single cell‐RNA‐seq in Opa1 cKO versus CT 4 weeks post‐TAM (Figure 3a; Figure S3A). From 4362 CT and 6296 Opa1 cKO cells, the top five genes of nine clusters are shown in the heatmap, and clusters were classified by known markers of brain cell types (Figure S3B) (Becht et al., 2018). Four clusters of non‐neuronal lineages were excluded from further study, including Cluster 3—ependymal cells (Ccdc153), Cluster 4—mural cells (Vtn), Cluster 7—endothelial cells (Cldn5), and Cluster 8—oligodendrocytes (Mbp) (Figure S3D) (Artegiani et al., 2017; He et al., 2016; Kalinina & Lagace, 2022; Liu et al., 2022; Mizrak et al., 2019). The 2847 CT and 3466 Opa1 cKO cells from the neuronal lineage were re‐clustered and the UMAP distribution yielded three distinct populations namely Cluster 0 that consists of Aldoc, Gfap, Sox2, and Id3 that define resident glia‐like NSCs (NSC pool); Cluster 1 that contains activated NSCs marked by Ascl1, and Cluster 2 that includes a mixed population of neural progenitors (Mki67 and Eomes), and differentiating neurons (Dcx, Sox4, Sox11, and Prox1) (Figure 3b; Figure S3C). A subset of cluster markers is highlighted in violin plots and heatmaps defining each cluster (Figure S3E,F). For example, Aldoc and ApoE are highly expressed in quiescent NSCs (Urban et al., 2019), both of which are over‐represented in Cluster 0 NSCs (Figure S3E,F). Ascl1 is an essential transcription factor for NSC activation, and its expression is induced specifically in Cluster 1 NSCs (Figure S3D,E) (Andersen et al., 2014). Dcx, a marker of immature neurons, is over‐represented in Cluster 2 (Figure S3D–F). Unbiased transcriptional profiling suggests that there is a metabolic change in Opa1‐deficient cells due to the decline in mitochondrial OXPHOS genes such as mt‐Nd1, mt‐Nd4, and mt‐Co1 (Figure 3c–e). We observed the activation of classic cellular stress response pathways, including the integrated stress response (ISR) pathway (Atf4, Chac1, Slc7a11, Slc3a2, Ddit3, and Ddit4) in all the clusters of neuronal lineages (Figure 3c–e). Notably, the resident quiescent NSCs (cluster 0) showed a prominent ISR led by ATF4 and its downstream targets (Figure 3c–e). These results suggest that the early onset of stress signaling in NSCs impairs their function. We tested this hypothesis in the process of neuronal differentiation. In Opa1 KO cells, 24% of mRNA is unspliced, as compared to 37% in CT controls, suggesting a lower level of new transcript synthesis (Figure 3h). RNA velocity revealed a preserved neurogenesis progression from proliferating cells to neurons in CT cells. However, the loss of Opa1 led to aberrant differentiation, suggesting an impairment in Opa1 cKO neuroblasts where arrows indicate a reverse trend to an undifferentiated state (Figure 3f). Impaired differentiation is manifested by decreased differentiation markers in Cluster 2 of Opa1 cKO cells. For example, RNA velocity of proliferation (Mki67 and Pcna) and differentiation (Eomes, Dcx, and Neurod1) markers showed a tendency to generate new transcripts in CT (more cells in green) but not in Opa1 KO (more cells in red) (Figure 3g). Our single‐cell analysis suggests that Opa1 loss results in an impairment in neurogenesis that begins in quiescent NSCs and reveals an induction of genes associated with the ISR pathway. We therefore examined its impact in adult NSC function under stress conditions.

### 3.4 Loss of Opa1 in NSCs induces the ISR as a result of mitochondrial dysfunction and oxidative stress

**FIGURE 4:**
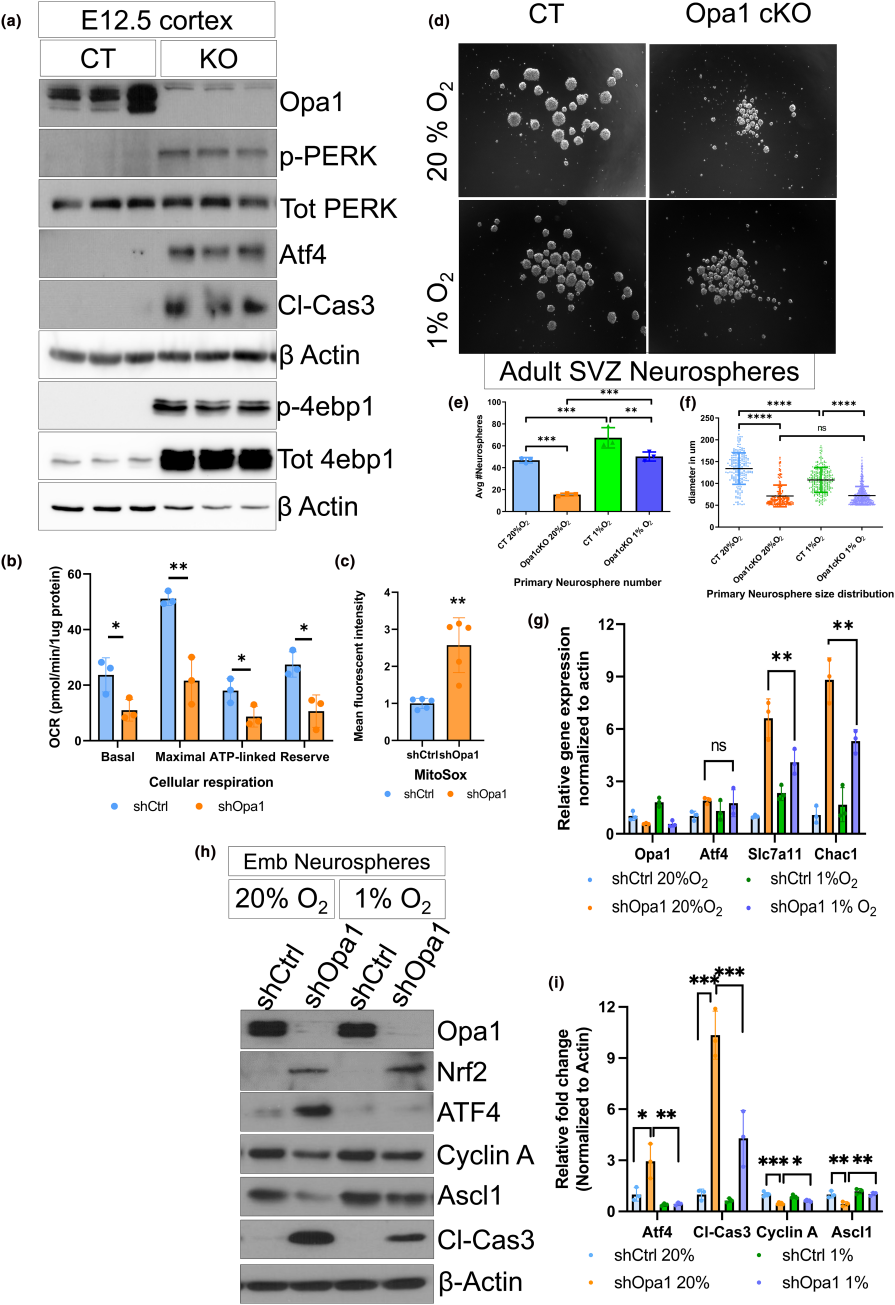
ATF4 pathway is activated by mitochondrial dysfunction and reductive metabolism under hypoxia resolves ATF4 activation. (a) Western blot of the ISR pathway‐related proteins in E12.5 embryonic cortex of CT and Opa1 KO post Opa1 deletion as mentioned in the plot. (b) Cellular oxygen consumption rate was measured using XF24 extracellular flux analyzer. Bar graphs represent the cellular respiration of basal, maximal, reserved, and ATP‐linked respiration between the listed conditions. *n* = 3 animals. (c) Normalized mean intensity of MitoSOX Red was calculated from live cells and plotted as bar graph. (d) Phase contrast images of neurospheres from Adult NSCs of CT and Opa1cKO animals in listed Oxygen exposure conditions. (e) Bar graph representing the average number of primary neurospheres formed in CT and Opa1 cKO neurospheres growing under normoxic and hypoxic conditions. *n* = 3 biological replicates; data are presented as mean ± SD (**p < 0.01, and ***p < 0.001, one‐way ANOVA). (f) Diameter size (in μm) of CT and Opa1 cKO neurospheres grown in hypoxic and normoxic conditions. 120–130 neurospheres measured with *n* = 3 biological replicates; data are presented as mean ± SD (****p < 0.0001, One‐way ANOVA). (g) RT‐qPCR results of stress response genes under hypoxic and normoxic conditions. *n* = 3 animals; data are presented as mean ± SD (**p < 0.01, One‐way ANOVA) (h) Representative western blot image from total protein lysates of embryonic neurospheres (E12.5) treated with LV‐shCtrl or shOpa1 and grown in normoxic and hypoxic conditions. (i) Western blot quantification of ATF4, cl‐Cas3, cyclin A, and Ascl1 in CT and Opa1 KO. Mean intensity was normalized to wild type in the bar graph. *n* = 5 animals; data are presented as mean ± SD (*p < 0.05, ** < 0.01 and *** < 0.001 One‐way ANOVA). CT, control transgenic; OPA1 cKO, OPA1 conditional knockout; shCtrl, Lentivirus vectors shRNA scramble control; shOPA1, Lentivirus vectors shRNA to OPA1.

To validate the induction of the ISR following impaired mitochondrial function, we first confirmed mitochondrial dysfunction upon Opa1 loss in our models and generated an embryonic model of BF‐1‐driven Opa1KO in NSCs. The embryonic model of Opa1 loss in NSCs recapitulated defects in neurogenesis observed such as decreased protein and gene expression of key activation and neurogenic markers, including Ascl1, Notch (N^ICD^), and Dcx (Figure S4A,B). Importantly, loss of Opa1 in embryonic NSCs also induced the same ISR as that observed in the adult Opa1cKO model (Figure 4a). Consistent with adult hippocampal cKO and in vitro knockdown of Opa1 (Figure 3d; Figure S4G,I), NSCs derived from the embryonic cortex also exhibited the activation of the ATF4 pathway, including increased ATF4 protein and activation of PERK, a known activator of the ISR (Figure 4a). It was also noted that there was an increase in the levels of the global translational repressor EIF4EBP1 (Figure 4a). The quantification of mitochondrial electron transport chain (ETC) proteins revealed a significant decrease in subunits representing complexes CI, CIII, and CIV in Opa1‐deficient developing cortex (Figure S4C,D). While ATP assays revealed no significant change in total ATP, there was a significant decrease in oligomycin‐sensitive OXPHOS‐derived ATP in cells from Opa1 KO cortex (Figure S4E). Furthermore, basal and ATP‐linked OCR are significantly reduced in Opa1 knockdown (KD) NSCs (Figure 4b). NAD^+^ measurements, as a readout of mitochondrial Complex I activity, revealed a significant decrease in total NAD^+^ levels and NAD+/NADH+ ratio in the Opa1 KD neurospheres (Figure S4F). MitoSOX flow cytometry analysis revealed a significant increase in mitochondrial ROS levels upon Opa1 KD (Figure 4c; Figure S4H). Similar to the OPA1 phenotype reported, mitochondrial dysfunction is observed in aging models (Figure S4J) (Beckervordersandforth et al., 2017) highlighting the crucial role of mitochondrial function in NSCs. Overall, these results confirm that Opa1 loss in NSCs results in OXPHOS impairment, decreased OCR, reduced mitochondrial ATP production, and elevated ROS levels.

To determine whether dysfunctional oxidative metabolism and redox stress are involved in the ISR induction and the impairment in NSC function, we asked if hypoxia (1% O_2_) could mitigate the ISR and rescue NSC function. Under normoxic culture conditions, the Opa1 cKO‐derived SVZ‐NSCs formed fewer and smaller neurospheres indicating a neurogenesis defect corroborated by our in vivo findings (Figure 4d–f; Figure S4G). On the other hand, significantly more neurospheres were produced under hypoxic conditions in Opa1cKO (Figure 4d–f; Figure S2C–E). These results suggest that impairment in NSC function and progenitor proliferation could at least partially be rescued once oxidative stress is resolved. Importantly, the incubation of shOpa1‐transduced neurospheres under hypoxia allowed ATF4 to revert to baseline levels (Figure 4h,i) and to downregulate the expression of downstream stress response genes, Slc7a11 and Chac1 (Figure 4g). Furthermore, this was accompanied by a partial rescue in proliferation (Cyclin A) and NSC activation (Ascl1) relative to shOpa1‐transduced neurospheres under normoxia (Figure 4h,i). In addition, Opa1 depletion increased levels of cleaved caspase 3 (cl‐Cas3) (Figure 4a,g), a cell death marker, which was rescued upon incubation in hypoxia and alleviation of oxidative stress (Figure 4h,i). NSC proliferation and activation defects measured by Cyclin A and Ascl1 were also rescued under hypoxic conditions, suggesting a partial restoration of NSC function (Figure 4h). Given the striking induction of the ATF4 pathway in NSCs following mitochondrial dysfunction, we next sought to investigate the role of ATF4 in preserving NSC survival and function under stress conditions.

### 3.5 ATF4 mediates a global stress response in NSCs to promote survival during mitochondrial stress

**FIGURE 5:**
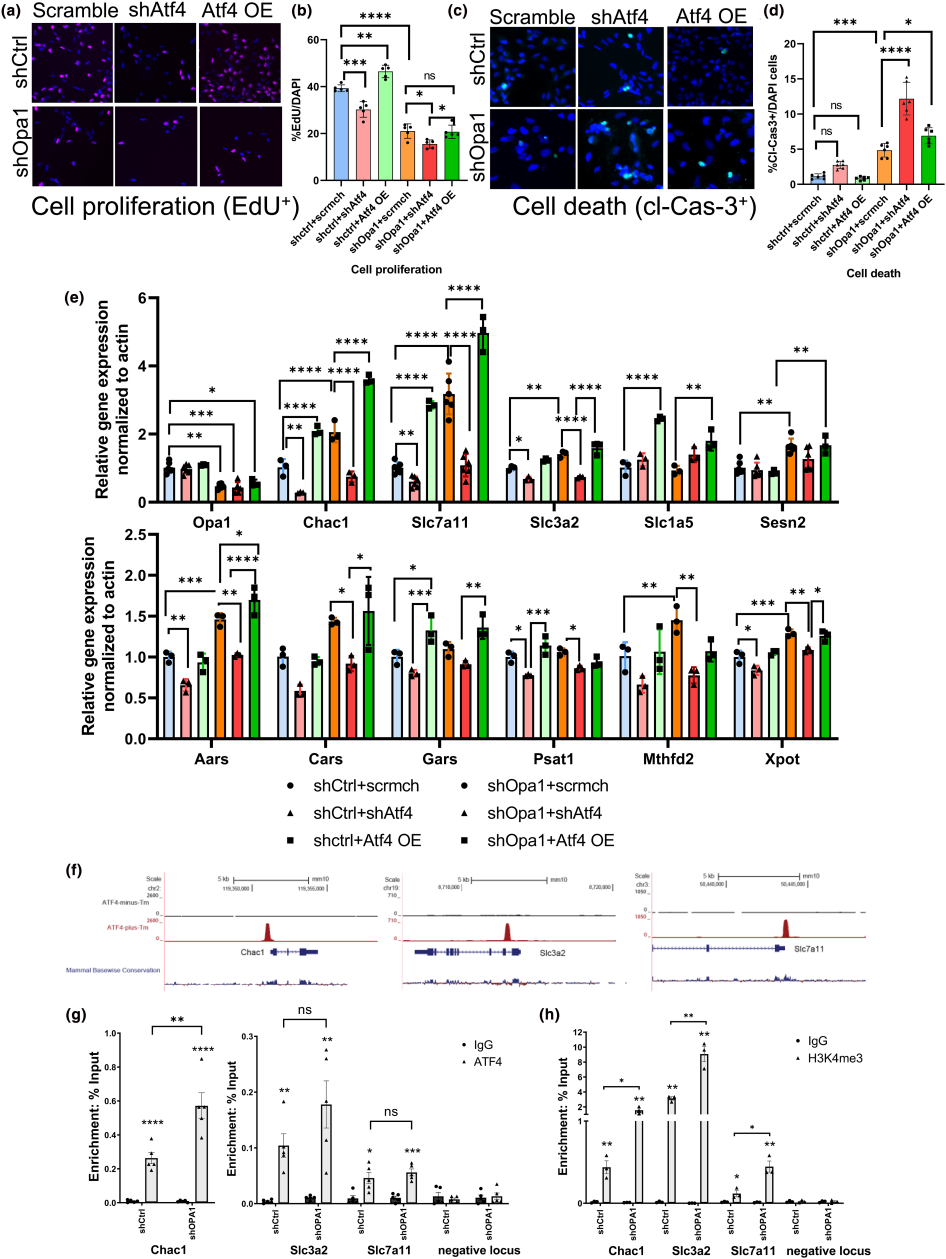
ATF4 function is required for cell proliferation and survival in normal and stressed state. (a) Representative images of EdU and DAPI staining in above mentioned conditions. Cell proliferation is measured using EdU+ cells normalized to total DAPI. (b) Quantification of the percent EdU+ over total DAPI+ cells represented in a bar graph. (c) Representative images of cleaved Caspase 3 (cl‐Cas‐3) and DAPI staining in abovementioned conditions. Cell death is measured using cl‐Cas3+ cells normalized to total DAPI. (d) Quantification of percent cleaved caspase 3+ over total DAPI+ cells represented in bar graph. *n* = 5–6 biological replicates; Data are presented as mean ± SD (*p < 0.05, **p < 0.01, and ***p < 0.001, ****p < 0.0001, One‐way ANOVA). (e) RT‐qPCR analysis in mentioned conditions for ATF4 targets involved in amino acid transport, tRNA aminoacylation, export, and one‐carbon metabolism. *n* = 3–6 animals; Data are presented as mean ± SD (*p < 0.05, **p < 0.01, and ***p < 0.001, One‐way ANOVA). (f) Schematics indicating the binding of ATF4 in mouse embryonic fibroblasts as identified through ChIP on Chac1, Slc3a2, and Slc7a11 genes (Han et al., 2013). (g) ATF4 ChIP from shCtrl and shOpa1 KD neurosphere. (h) H3K4me3 ChIP from shCtrl and shOpa1 KD neurosphere. *n* = 3–6 animals; data are presented as mean ± SEM (*p < 0.05, **p < 0.01, and ***p < 0.001, 2‐tailed Student's *t* test). ATF4 OE, ATF4 overexpression vector; scrmch, Lentivirus vectors shRNA scramble mCherry; shATF4, Lentivirus vectors shRNA to ATF4; shCtrl, Lentivirus vectors shRNA scramble control; shOPA1, Lentivirus vectors shRNA to OPA1.

To investigate the function of the ATF4‐ISR pathway in NSCs, we manipulated ATF4 expression levels in control and Opa1 KD NSCs (Figure S5A). Under control conditions, EdU incorporation revealed that ATF4 KD results in decreased proliferation while ATF4 overexpression increases the rate of proliferation (Figure 5a,b). In Opa1 KD cells, where ATF4 is already at elevated levels, ATF4 KD further decreased cell proliferation (Figure 5a,b). In a similar manner, ATF4 KD under Opa1‐deficiency exacerbated cell death (Figure 5c,d). These results indicate the importance of ATF4 in maintaining survival and proliferation of NSC under mitochondrial stress conditions.

To identify ATF4 downstream targets that may be mediating these protective effects, we quantified potential ATF4 target genes by RT‐qPCR (Figure 5e). Our results reveal a number of stress response genes that are responsive to ATF4 manipulation, including genes required for amino acid transport (Slc7a11, Slc3a2, and Slc1a5) and tRNA charging (Aars, Cars, and Gars) among other genes such as Chac1, Mthfd2, and Xpot. Our ATF4 ChIP‐qPCR showed a significant increase in ATF4 binding at the transcription start sites (TSS) of Chac1, Slc3a2, and Slc7a11 under Opa1 KD (Figure 5f,g). Histone H3 lysine 4 trimethylation (H3K4me3), which correlates with open chromatin (active transcription), was significantly more enriched at those loci, consistent with increased transcription (Figure 5h).

### 3.6 Slc7a11 and xCT system are required for NSC function and survival under ATF4‐inducing stress

**FIGURE 6:**
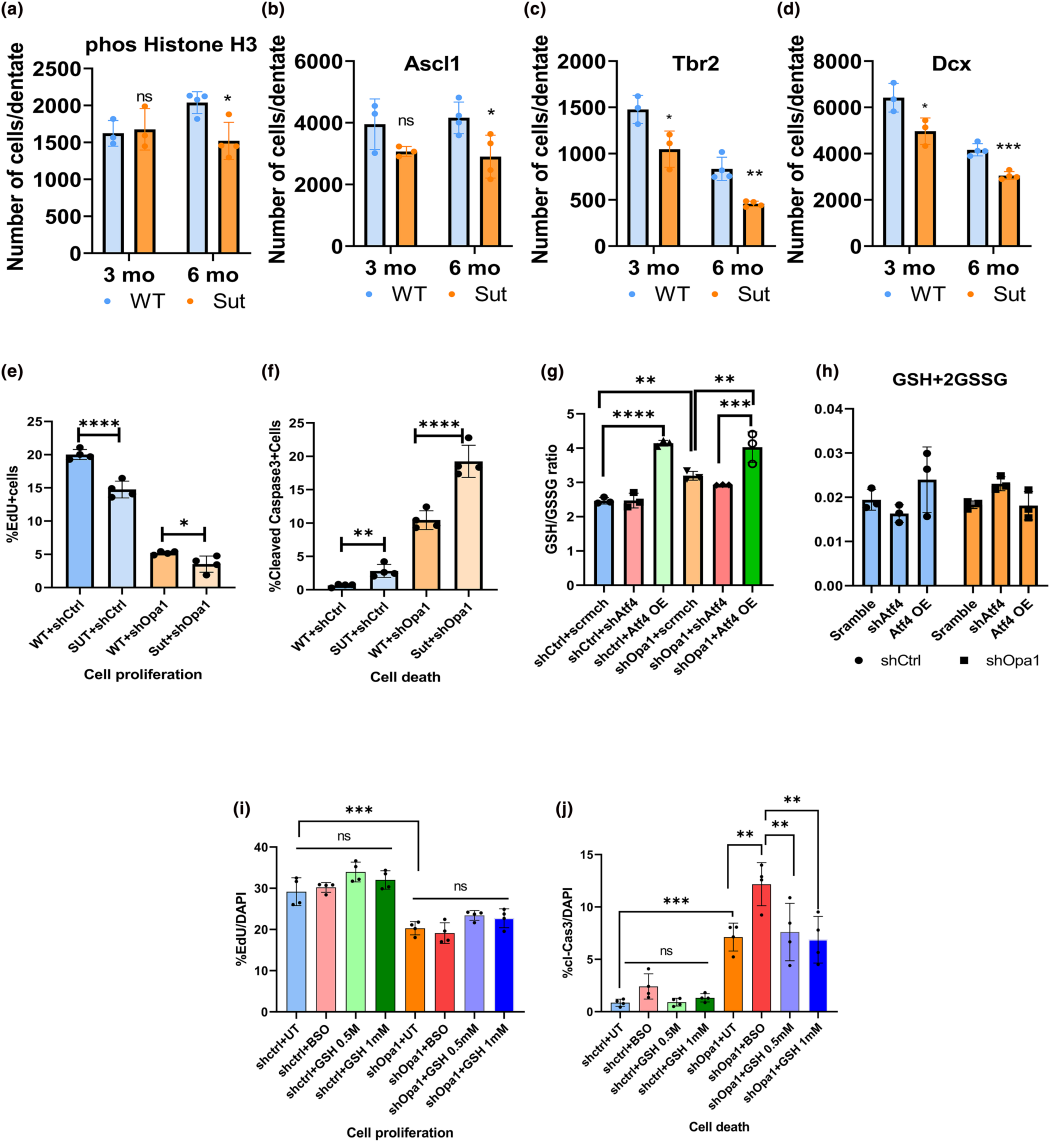
Slc7a11, a key target of ATF4, and glutathione redox are required for NSC function and survival. (a–d) Histological analysis of phospho‐Histone 3(Proliferation), Ascl1(Activation), Tbr2(TAP), and Dcx(Newborn neurons) in 3 months and 6 months old wild‐type and sut/sut adult mice. *n* = 4–5 animals; data are presented as mean ± SD (*p < 0.05, **p < 0.01, and ***p < 0.001, Student's *t* test). (e) In vitro monolayer culture of WT and Sut mice infected with scramble and shOpa1. Bar graph for percent EdU+ over total DAPI+ cells and (f) Cleaved caspase 3+ over total DAPI+ cells. Data are presented as mean ± SD (*p < 0.05, **p < 0.01, and ***p < 0.001, Student's *t*test). (g, h) Glutathione measurement using HPLC of GSH:GSSG ratio and total GSH levels in embryonic neurospheres in mentioned conditions, *n* = 3 animals; (i) Quantification of percent EdU+ over total DAPI+ cells represented in bar graph for the mentioned conditions. (j) Quantification of percent cleaved caspase 3+ over total DAPI+ cells represented in bar graph. *n* = 3–4 animals; data are presented as mean ± SD (*p < 0.05, **p < 0.01, ***p < 0.001 and ****p < 0.0001, one‐way ANOVA).

As Slc7a11 is a prominent target for ATF4 and is highly induced under stress conditions, we asked whether ATF4‐mediated induction of Slc7a11 plays a critical role in mediating cellular adaptation to stress through regulation of redox balance. xCT system is a heterodimeric transmembrane channel that consists of Slc7a11 and Slc3a2 important for cystine and glutamate exchange (Lim et al., 2019). We utilized Slc7a11 mutant mice (Sut) and WT littermates to determine if dysfunctional Slc7a11/xCT impacts adult hippocampal neurogenesis at 3 and 6 months of age. Sut mice showed a significant decrease in NSC proliferation (phospho‐Histone 3) and activation (Ascl1) at 6 months of age (Figure 6a,b), in addition to a decrease in TAP cells (Tbr2) and newborn neurons (Dcx) at both 3 and 6 month time points (Figure 6c,d). Similarly, neurosphere assay in vitro showed reduced neurosphere formation, decreased NSC proliferation (EdU) and increased cell death (cl‐Cas3) in Sut mice compared to controls (Figure S6a,6e,f). Importantly, under Opa1 KD, the Sut‐derived NSCs were more sensitive to mitochondrial dysfunction‐induced cell death when compared to WT cells (Figure 6f). These results suggest an important role of Slc7a11 in maintaining NSC survival and proliferation in basal and stressed conditions.

Consistent with ATF4 function in the regulation of glutathione production (Torrence et al., 2021), we further tested the impact of ATF4 pathway manipulation on the glutathione levels in the NSCs. GSH:GSSG ratio increased in Opa1 KD under ATF4 overexpression conditions (Figure 6g), while total GSH levels remained similar under all conditions (Figure 6h). These data suggest that ATF4 plays an important role in regulating glutathione redox balance, possibly through ATF4‐downstream target Slc7a11. To test the role of GSH in NSC under Opa1 KD, we added exogenous reduced GSH or depleted the pool with buthionine sulfoximine (BSO). We observed no significant effect on NSC proliferation (EdU+cells) in shOpa1‐KD cells compared to controls (Figure 6i). However, the depletion of the GSH pool exacerbated cell death in shOpa1‐KD cells (Figure 6j). Together, these findings demonstrate the critical role of ATF4 and its downstream target Slc7a11 in mediating the protection of NSC under mitochondrial dysfunction.

## 4 DISCUSSION

In this report, we reveal a novel mechanism by which neural stem and progenitor cells adapt to mitochondrial dysfunction, typically found in aging and neurodegeneration. We show that mitochondrial dysfunction leads to a decline in neurogenesis due to impaired NSC activation and identify the ATF4 pathway as a critical mediator to enable cellular adaptation to stress. We show that ATF4 preserves NSC proliferation and survival under stress by mediating glutathione production through the activation of Slc7a11 as a regulatory target.

We, and others, have shown that the proper maintenance of mitochondrial dynamics and metabolism is essential for adult neurogenesis, which impacts learning and memory (Beckervordersandforth et al., 2017; Iwata et al., 2020; Khacho et al., 2016). Our previous studies have shown that NSC differentiation is reliant on a metabolic switch from glycolysis to OXPHOS, whereby mitochondrial signaling is essential for neurogenesis (Khacho et al., 2016, 2017, 2019). Here we show that metabolic changes due to Opa1 deficiency impacts all stages of neurogenesis, from quiescence to activation, differentiation, and survival through transcriptomic alteration and induction of the ISR (Inak et al., 2021) (Figures 1 and 3). While the number of NSCs was not different at 4 weeks post TAM in vivo (Figure 1h), our sc‐RNA‐seq data clearly show a perturbation in early NSCs manifested by the induction of the ISR, including the induction of ATF4 and its targets (ATF4, Slc7a11, Chac1 and Xpot; Figure 3d), which may explain the impairment in NSC activation (Figure 2l). This is in line with the observed early stress signaling in the SVZ FACS‐sorted cells at 10 days post‐TAM (Figure 1i; Figure S1D).

In addition to the activation of the ISR, mitochondrial dysfunction leads to transcriptomic alterations in markers of neuronal fate decision such as Ascl1, Dcx, Sox4, Sox11, and Prox1 (Figure 3c; Figures S1D and S4A). Similar impairments in NSC function were observed in Alzheimer's disease, where hypo‐maturation and dedifferentiation have been reported (Mertens et al., 2021). Together, these studies support defective differentiation and neurogenesis in which mitochondrial dynamics is playing a central role.

To ask if oxidative stress is the key mechanism underlying NSC impairments, we subjected Opa1‐deficient cells to hypoxia to enable cells to adapt to OXPHOS‐independent energy sources and thereby lower oxidative stress. We show that hypoxia can rescue NSC defects, including proliferation, expression of differentiation/activation genes (Ascl1), mitigation of ATF4 expression, and cell survival (Figure 4h). These findings demonstrate that oxidative stress is a key mediator of the ISR in Opa1‐deficient NSCs and that the neurogenesis defect can be rescued when mitochondrial oxidative stress is resolved through appropriate treatments.

Our results reveal compelling evidence that the ISR plays a critical role in the adaptation to Opa1‐mediated stress in embryonic and adult NSCs. The ISR is a key adaptive mechanism to counteract different cellular stress conditions, including protein misfolding, nutrient deprivation, oxidative stress, and restoring metabolic and protein homeostasis (Costa‐Mattioli & Walter, 2020). Our study shows that Opa1 deficiency‐induced mitochondrial dysfunction triggers an ISR response and leads to upregulation of ATF4, as has been described in cell lines (Fessler et al., 2020; Guo et al., 2020; Quirós et al., 2017). Previously, the ISR was found to mitigate cardiomyopathy disease pathology induced by OXPHOS dysfunction in the heart (Ahola et al., 2022); however, little is known regarding how the ISR impacts NSC function and survival in response to mitochondrial dysfunction. We show that the ATF4 pathway enables NSCs to adapt to stress and survive, and that this can be rescued by providing a hypoxic environment, which results in a suppression of ATF4 induction and its downstream targets. Under these conditions, Opa1‐deficient NSCs could proliferate and survive by rewiring their energetic pathways. Previous studies with cord blood hematopoietic stem cell (HSC) revealed that the upregulation of ATF4 is important for HSC survival under basal and nutrient deprivation conditions (van Galen et al., 2018). However, we demonstrate that ATF4 and the ISR instigate a protective mechanism enabling NSCs to adapt to mitochondrial dysfunction stress.

ATF4 induction activates the transcription of a series of downstream targets in both stimulated and stressed conditions (Torrence et al., 2021), and the cellular state dictates the dichotomous function of ATF4. For example, at basal levels without stress, ectopic ATF4 expression leads to cell growth and proliferation. However, the presence of ATF4 under stress (by stimuli or overexpression) enhances survival (Figure 5b,d). Previous results suggest that ATF4 in control conditions increases cell proliferation through its targets that mimic a growth signal and induce an anabolic program (Torrence et al., 2021). In contrast, in the context of mitochondrial dysfunction, ATF4 activation triggers a stress response essential to sustain survival.

While ATF4 has multiple targets, it remains unknown which target is most critical in maintaining the survival of adult NSCs under mitochondrial dysfunction and metabolic stress. Slc7a11 mutation has been shown to alter regional, cellular, and subcellular mouse brain morphology, and induce hyperexcitable neurons following the administration of chemoconvulsants (pentylenetetrazole) (Sears et al., 2021). Furthermore, in a mouse model of ataxia (Kcna1‐KO), exhibiting enlarged hippocampus and aberrant adult neurogenesis, the levels of Slc7a11 mRNA are increased. In this context, Kcna1‐Slc7a11 double knockout has been shown to decrease aberrant neurogenesis quantified by Dcx (Aloi et al., 2022). In our model, Slc7a11, a key target for ATF4, is highly induced in Opa1‐deficient NSCs in vivo and in vitro. Our results suggest that the depletion of GSH levels increases the vulnerability of Opa1 depleted NSCs (Figure 6). This is in line with previous reports where blocking Slc7a11 by the pharmacological agent, erastin, was shown to deplete GSH levels and mediate the stress response in immortalized tumor cell lines resulting in ferroptosis (Yang et al., 2014). In muscle stem cells, Opa1 was shown to regulate the expression of Slc7a11 and other genes involved in glutathione synthesis (Baker et al., 2022). Our data show that under basal condition, Slc7a11 plays a crucial role in adult hippocampal neurogenesis, as loss of Slc7a11 function impairs hippocampal neurogenesis by reducing NSC proliferation and neurogenesis (Figure 6a–d). We also show that Slc7a11 deficiency exacerbated cell death in NSCs in vitro following Opa1 knockdown (Figure 6f), suggesting the importance of the Slc7a11 and glutathione redox in NSC function under stress.

In summary, we show that mitochondrial dysfunction typically found in neurodegenerative diseases (through Opa1 loss) leads to neurogenesis impairment which subsequently causes learning and memory deficits. Opa1 loss alters the course of NSC progression by impairing proliferation and neurogenesis confirming the essential role of mitochondrial function. We identify ATF4 and its downstream target Slc7a11 as major players in ROS defense and survival of NSCs under Opa1 deficiency.

### 4.1 Limitations of the study

The majority of the work relies on a transgenic model of Opa1 loss to study mitochondrial dynamics. Thus, in the future, other mitochondrial proteins such as Mfn1/2 and Drp1 should be manipulated in order to get a better understanding of the role of mitochondrial dynamics in neurogenesis. The investigation of the role of ATF4 is limited to in vitro studies and a manipulation of ATF4 pharmacologically or through gene editing in the adult SGZ brain would be helpful. Given the lower number and size of neurospheres formed from SGZ cell culture compared to SVZ and the limited number of passages, most of the in vitro ATF4 knockdown and overexpression was performed in embryonic or SVZ neurospheres. Future work should focus more on the role of ATF4 and Slc7a11 in the adult SGZ niche.

## AUTHOR CONTRIBUTIONS

Conceptualization and methods: M.A.I, M.B., Y.L., B.C.F., I.C., S.P., ME.H., M. Khacho., and R.S.S. Investigation, M.A.I., V.J., B.C.F., M.B., I.C., Y.L., S.P., J.C., S.W., and M.Kannan. Resources: ME.H., M.Khacho, and R.S.S. Writing: M.A.I., M.B., S.P., Y.L., I.C., S.W., M.Khacho., and R.S.S. Review, all authors.

## FUNDING INFORMATION

This work was supported by grants from the CIHR foundation grant to RSS GR000324(154309); CIHR to M. Khacho (438989), and Canada Research Chair (CRC) to M. Khacho. We also thank Mito2i fellowship to MAI.

## CONFLICT OF INTEREST STATEMENT

The authors declare no conflict of interest.

## Supporting information


Figures S1–S6

Table S1

Table S2

## Data Availability

Data have been deposited in GEO under accession number GEO: GSE233461 and GSE233464. The published article includes all datasets generated or analyzed during this study and no new code was produced. Any additional information required to reanalyze the data reported in this paper is available from the lead contact upon request.
