# Supplementary material for: The integrated stress response promotes neural stem cell survival under conditions of mitochondrial dysfunction in neurodegeneration
Source: Aging Cell. 2024 May 16;23(7):e14165. doi: 10.1111/acel.14165 (PMC11258489; doi:10.1111/acel.14165)
Supplement: Supplementary file 1 — Figures S1–S6 [file ACEL-23-e14165-s002.pdf]

Supplementary figure 1

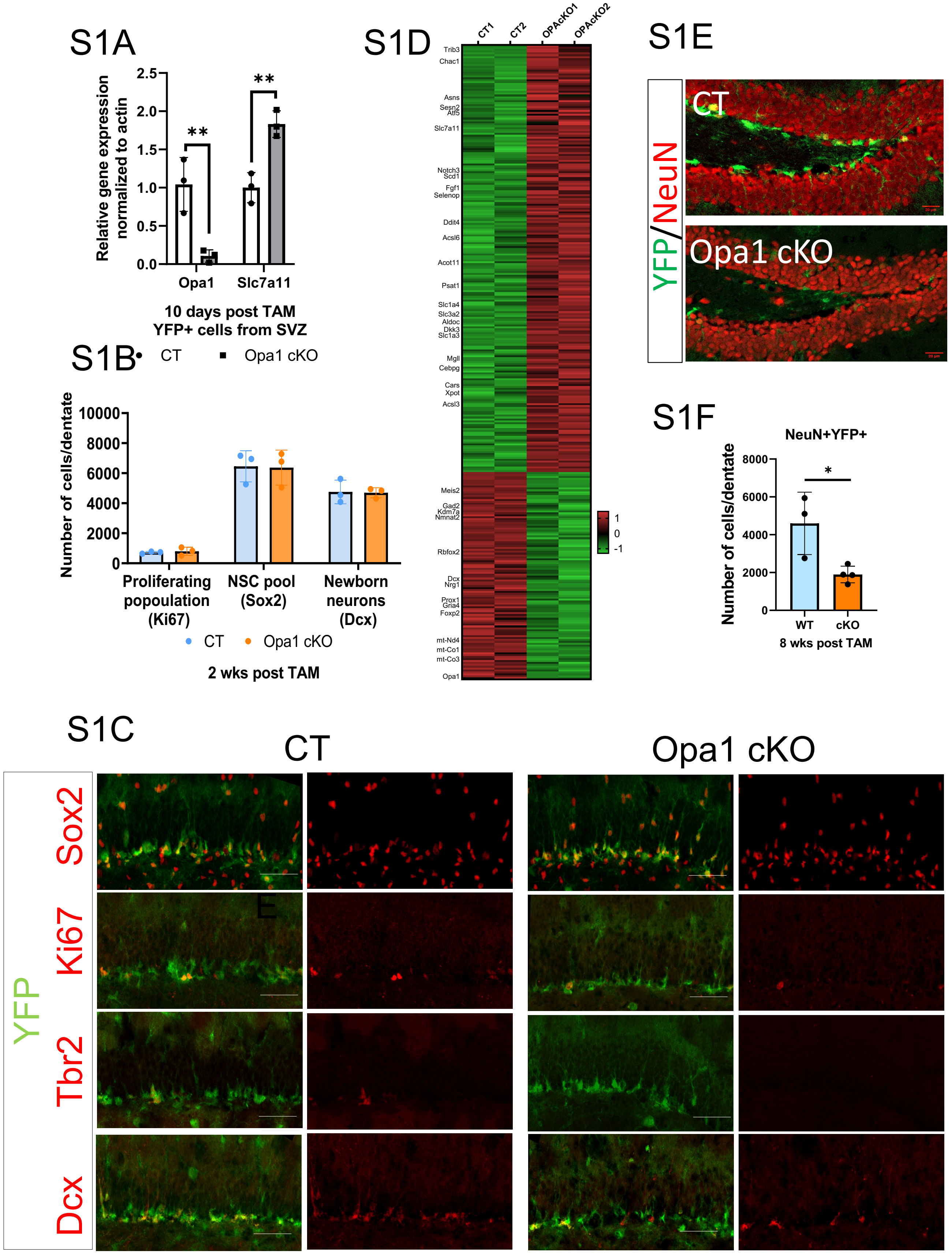

Supplementary figure 2

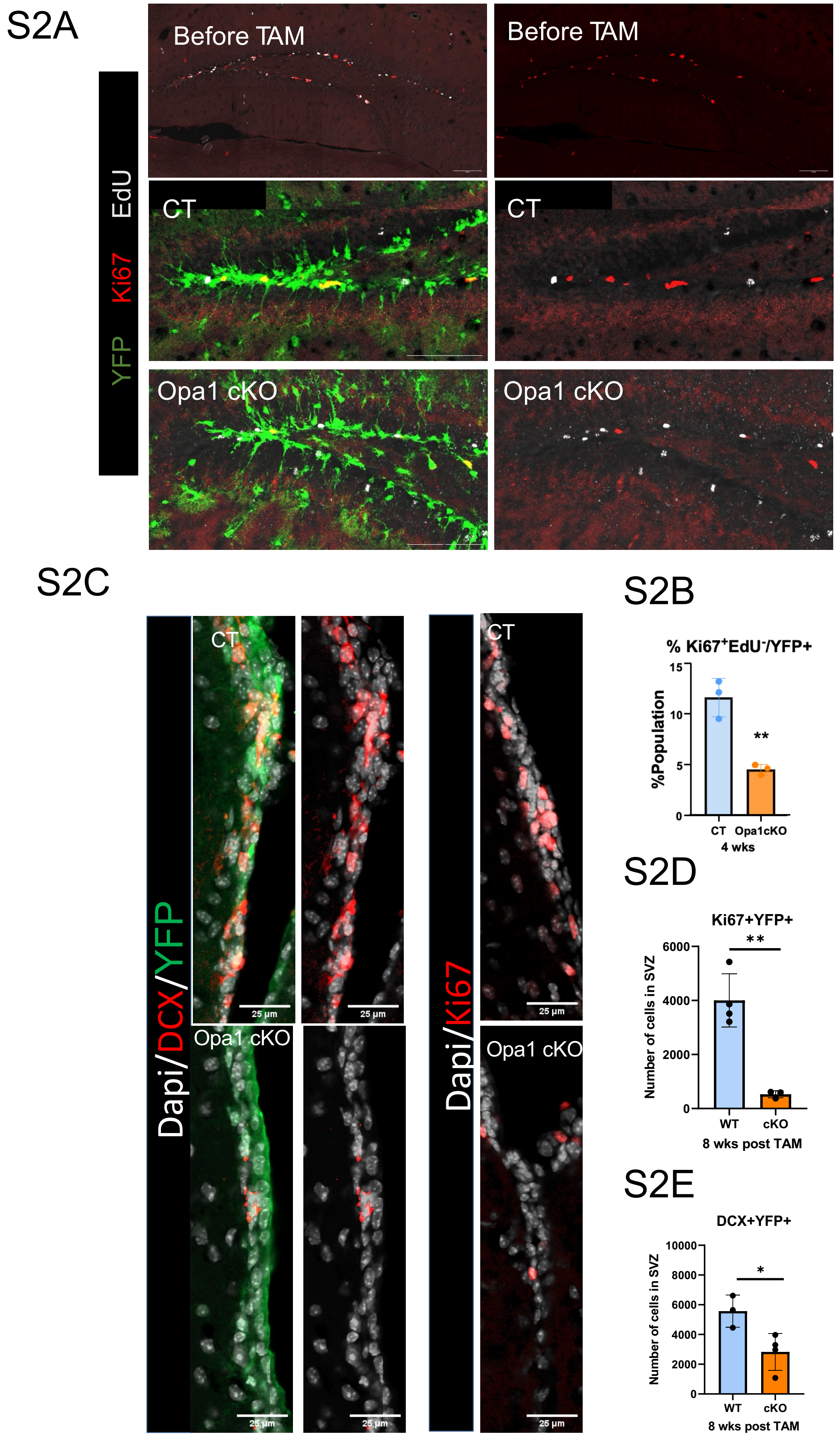

Supplementary figure 3

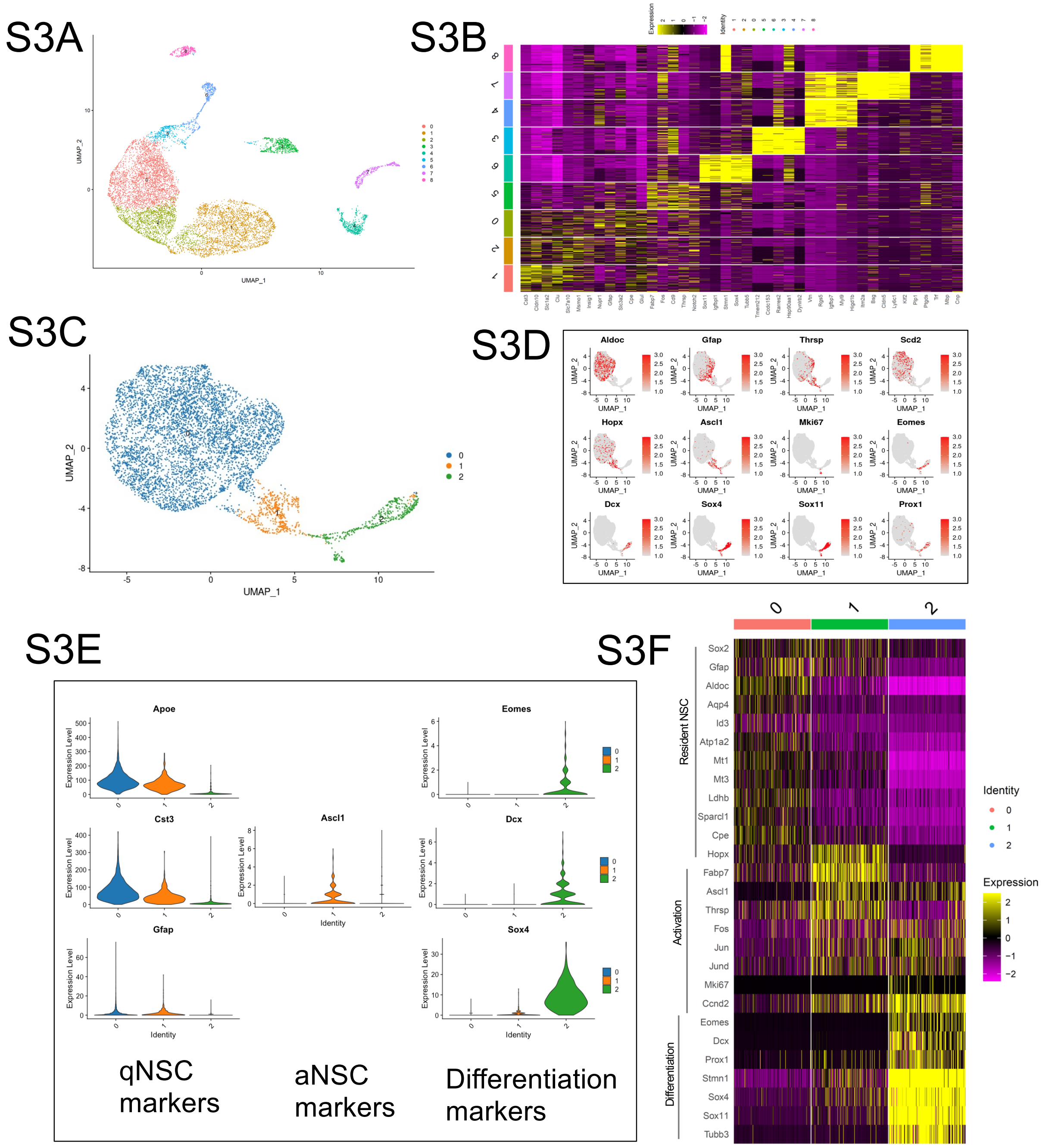

Supplementary figure 4

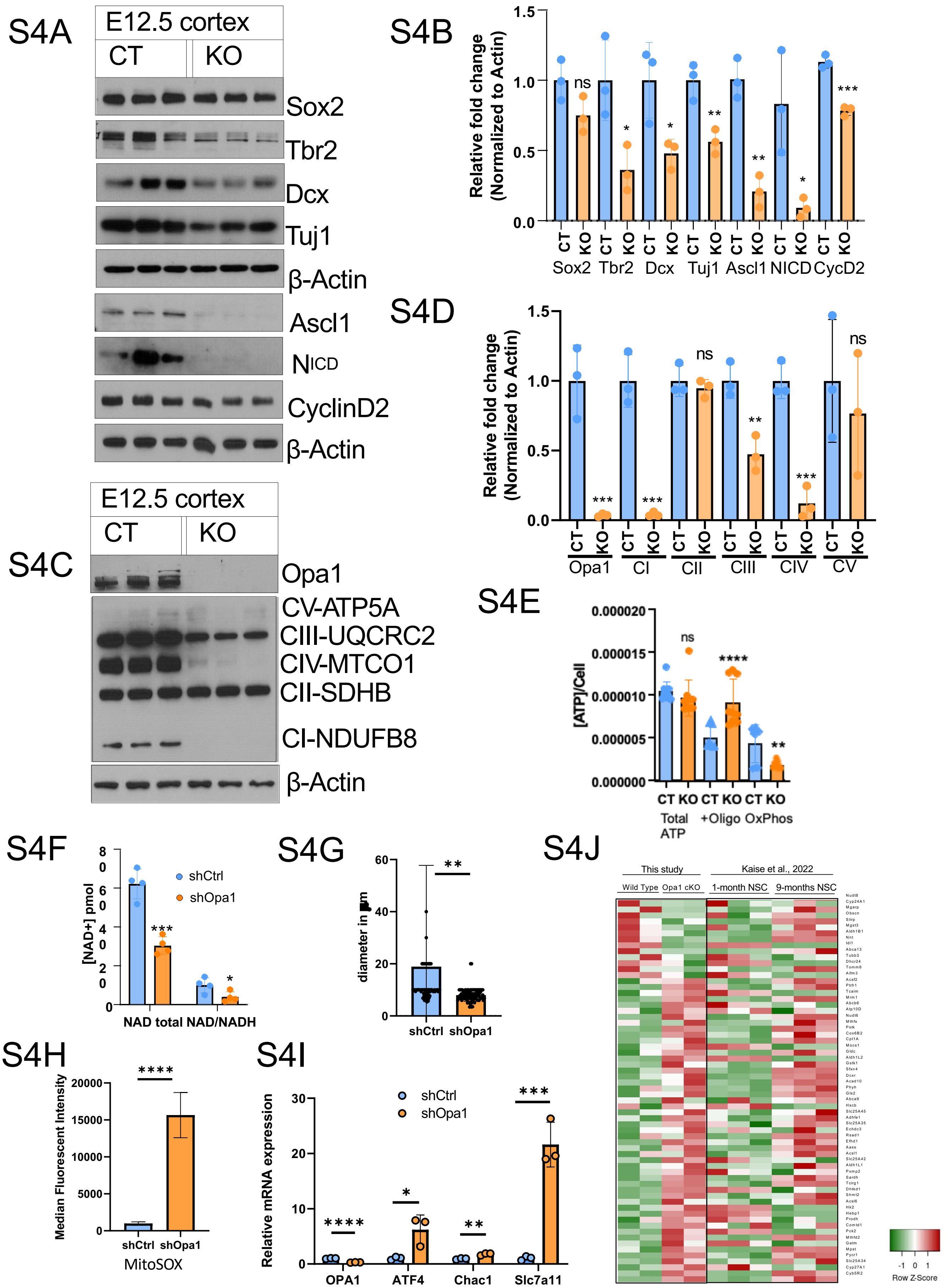

Supplementary figure 5

S5A

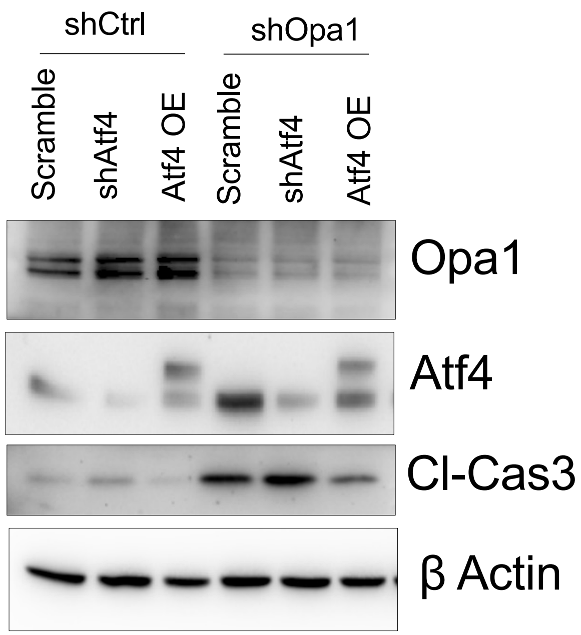

Supplementary figure 6

S6A

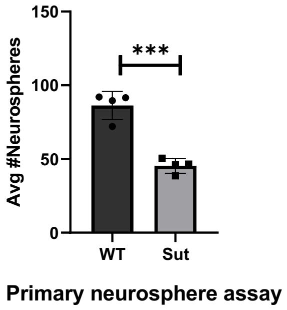

Figure 1 supplementary: Opa1 loss stalls NSC proliferation and activation in adult dentate gyrus.

S1A) qPCR analysis of YFP sorted cells from adult SVZ of Opa1 and Slc7a11, normalized to β Actin. (n = 3 animals); Data are presented as mean ± SD, (*$p < 0.05$, **$p < 0.01$, and ***$p < 0.001$, student's t-test). **S1B)** Bar graphs of the total number of cells per dentate in CT and Opa1 cKO 2weeks post Tamoxifen administration. **S1C)** Representative IHC images of Sox2, Ki67, Tbr2 and Dcx (red) colabelled with YFP (green) (Scale bar = 50 µm). (n = 3 animals); Data are presented as mean ± SD **S1D)** Heatmap of YFP sorted cells from adult SVZ of significant upregulated and downregulated genes (relative gene expression range from green -1 to red 1). S1E) Representative IHC images of NeuN (Red), colabelled with YFP (green) (Scale bar = 20 µm). S1F) Bar graphs of total number of cells per dentate in CT and Opa1 cKO 8 weeks post Tamoxifen administration. (n = 3-4 animals); Data are presented as mean ± SD, (*$p < 0.05$, student's t-test).

Figure 2 supplementary: Opa1 loss impacts NSC proliferation and activation.

S2A) Representative image for $Ki67^+$ cells and EdU label before TAM and at 4 weeks post TAM in CT and Opa1 cKO sections (Scale bar = 100 µm). **S2B)** Percentage $Ki67^+$ EdU^- cells out of the YFP^+ 4 weeks post-TAM in the dentate gyrus. (n = 3 animals); Data are presented as mean ± SD, (**$p < 0.01$, student's t-test). **S2C)** Representative image for YFP+ (green), Dcx+ (red), Ki67+ (red) and DAPI (white) in the SVZ 8 weeks post-TAM in CT and Opa1 cKO sections (Scale bar = 25µm). **S2D-E)** Bar graphs of total cells per SVZ in CT and Opa1 cKO 8 weeks post-TAM administration. (n = 4 animals); Data are presented as mean ± SD, (*$p < 0.05$, **$p < 0.01$, student's t-test).

Figure 3 supplementary: Single-cell RNA-Seq suggests Opa1 deletion results in neurogenesis defect.

S3A) Integrated UMAP projection of CT and Opa1 cKO cells using Seurat into nine cell clusters based on their unique gene expression profiles. **S3B)** Heatmap demonstrating genes representative of each cluster. **S3C)** UMAP projections demonstrating curated clusters representative of neurogenesis. **S3D)** UMAP projections of cluster markers highlighting genes representative of each cluster, reflecting cluster-specific expression. **S3E)** The violin plots suggest the distinct pattern of cluster-specific gene expression. **S3F)** Heatmap of curated genes representative of different stages of neurogenesis.

Figure 4 supplementary: Opa1 loss leads to severe mitochondrial and NSC defects.

S4A&B) Western blot and quantification of neurogenesis markers Sox2, Tbr2, Dcx, Tuj1, Ascl1, N^{ICD} and Cyclin D2 in E12.5 embryonic cortex of CT and Opa1 KO. **S4C&D)** Western blot and quantification of mitochondrial complex subunits in E12.5 embryonic cortex of CT and Opa1 KO post Opa1 deletion as mentioned in the plot. Mean intensity was normalized to wildtype in the bar graph. n=3 animals; Data are presented as mean ± SD (*p < 0.05, **p < 0.01, and ***p < 0.001, Student's t-test). **S4E)** Quantification of total ATP using Cell titre-Glo assay from the embryonic cortex of BF1-Cre negative (Wildtype) and BF1 Cre positive Opa1 F/F mice on E12.5 days. n=3 animals; Data are presented as mean ± SD (*p < 0.05, **p < 0.01, and ***p < 0.001, Student's t-test). **S4F)** Total NAD+ and NAD+/NADH+ ratio from Control and Opa1 knockdown neurospheres. Values were normalized to the protein content of the cell lysate. **S4G)** The diameter of primary neurospheres formed in scrambled shCtrl and shOpa1 KD SGZ neurospheres culture after 10 days *in vitro*. Data are presented as mean ± SD (**p < 0.01, Student's t-test). **S4H)** Median intensity of MitoSOX Red was calculated from live SGZ cultured neurospheres and plotted as a bar graph. n= 5 biological replicates; Data are presented as mean ± SD (****p < 0.0001, Student's t-test. **S4I)** qPCR analysis of dissociated adult SGZ stem and progenitor cells neurosphere culture

transduced with scrambled shCtrl and shOpa1, normalized to β Actin. (n = 3 animals); Data are presented as mean ± SD, (*p < 0.05, **p < 0.01, ***p < 0.001, and ****p < 0.0001 student's t-test). **S4J)** Heatmap of selected mitochondrial genes from the SVZ RNA-seq compared to 1- and 9-month-old NSC RNA-seq data by Kaise et al., 2022. Color density indicates the z-score computed from the normalized read counts.

Figure 5 supplementary: ATF4 is required for cell proliferation and survival in normal and stressed states.

S5A) Representative Western blot images of Opa1, ATF4, Cl-Cas3, and β Actin in E12.5 embryonic neurospheres under the mentioned conditions.

Figure 6 supplementary: Slc7a11, a key target of ATF4, and glutathione redox are required for NSC function and survival. S6A) Quantification of number of primary neurospheres from adult SVZ of WT and Sut mice. (n = 4 animals); Data are presented as mean ± SD, (***p < 0.001 student's t-test).
