# Supplementary material for: The integrated stress response promotes neural stem cell survival under conditions of mitochondrial dysfunction in neurodegeneration
Source: Aging Cell. 2024 May 16;23(7):e14165. doi: 10.1111/acel.14165 (PMC11258489; doi:10.1111/acel.14165)
Supplement: Supplementary file 2 — Table S1 [file ACEL-23-e14165-s001.docx]

Supplementary Table 1:

| REAGENT or RESOURCE | SOURCE | IDENTIFIER |
| --- | --- | --- |
| Antibodies | | |
| IgG (Rabbit; ChIP) | Sigma-Aldrich | CAT#I8140 |
| β-actin-HRP (Mouse; WB) | Santa Cruz | CAT#sc-47778 |
| Atf 4 (D4B8) Rabbit mAb | Cell Signaling | CAT#11815S |
| Dcx (Rabbit; WB) | Cell Signaling | CAT#4604 |
| Mash1 (Mouse; WB) | BD Biosciences | CAT#556604 |
| GFP (Chicken; IHC) | Abcam | CAT#ab13970 |
| Nrf2 (Rabbit; WB) | Abcam | CAT#ab62352 |
| Cyclin A | Santa Cruz | CAT#SC596 |
| Cyclin D2 | Santa Cruz | CAT#SC593 |
| Opa1 | Abcam | CAT# AB42364 |
| Total OXPHOS Rodent WB Antibody Cocktail | Abcam | CAT#ab110413 |
| 4EBP1 | Cell Signaling | CAT#9644 |
| Phospho-4EBP1 | Cell Signaling | CAT#2855S |
| Sox2 (Goat; IHC) | Santa Cruz | CAT#sc-17320 |
| Sox2 (Goat; IHC) | Neuromics | CAT#GT15098 |
| ASCL1 (Rabbit; IHC) | Abcam | CAT#ab211327 |
| Ki67 (Rabbit; IHC) | Cell Marque | CAT#275R |
| Tbr2 (Rat; IHC) | Invitrogen | CAT#14-4875-82 |
| Dcx (Goat; IHC) | Santa Cruz | CAT#SC-8066 |
| Dcx (Guinea Pig; IHC) | Millipore | CAT#AB2253 |
| Dcx (Rabbit; IHC) | Cell Signaling | CAT#4604S |
| Hopx (Rabbit; IHC) | ATLAS Antibodies | CAT#HPA030180 |
| Eomes (Rat; IHC) | eBioscience | CAT# 14-4875-82 |
| mCherry (Rat; IHC) | ThermoFisher | CAT#M11217 |
| Active Caspase 3 (Rabbit; WB) | Cell Signaling | CAT#9664S |
| NeuN (Rabbit; IHC) | Cell Signaling | CAT#24307S |
| TOM20 (Rabbit; IHC) | ProteinTech | CAT#11802-1-AP |
| Nestin (Goat; IHC) | R&D systems | CAT#AF2736 |
| Chemicals, Peptides, and Recombinant Proteins |  |  |
| Tamoxifen | Millipore Sigma | CAT#T5648 |
| EdU | BaseClick | CAT#BCK647-IV-IM-M |
| Paraformaldehyde | E COM | CAT#Px0055-3 |
| Tissue-Tek OCT Compound | VWR | CAT#25608-930 |
| DAPI | Millipore Sigma | CAT#D9542 |
| Epredia™ Immu-Mount™ | Fisher | CAT#9990412 |
| Percoll | Millipore Sigma | CAT#GE17-0891-02 |
| DMEM/F12 | Gibco | CAT#11330-032 |
| DMEM/F12 no phenol | Invitrogen | CAT#11039-021 |
| B27 without Vitamin A | Gibco | CAT#12587010 |
| bFGF | Millipore Sigma | CAT#F-0291 |
| EGF | Millipore Sigma | CAT#E-1257 |
| heparin | Millipore Sigma | CAT#H-3149 |
| Penicillin/Streptomycin | Gibco | CAT#1570-063 |
| Matrigel | VWR | CAT#CACB356230 |
| B27 with Vitamin A | Gibco | CAT#17504044 |
| N2 | Gibco | CAT#17502048 |
| GlutaMAX | Gibco | CAT#35050061 |
| ACSF | N/A | N/A |
| HI-FBS | Wisent | CAT#080-150 |
| Clarity Enhanced chemiluminescence Western Blotting Substrate | Bio-Rad | CAT#1705061 |
| Papain | Cedarlane | CAT#LS003126 |
| DNase I | Roche | CAT#11284932001 |
| RNaseZAP | ThermoFisher | CAT#AM9780 |
| Critical Commercial Assays |  |  |
| Arcturus® PicoPure® RNA Isolation Kit | ThermoFisher | CAT#KIT0204 |
| Rotor-Gene SYBR® Green PCR Kit | Qiagen | CAT#204074 |
| SMART-Seq® v4 Ultra® Low Input RNA Kit for Sequencing | Takara | CAT#634890 |
| Nextera XT DNA Library Preparation Kit | Illumina | CAT#FC-131-1024 |
| Deposited Data |  |  |
| Sc-RNAseq | GEO Submission | GSE233465 |
| Bulk-RNA-Seq | GEO Submission | GSE233465 |
| Micro-dissected and FACS-isolated SVZ YFP+ NSCs | N/A | N/A |
| Micro-dissected SVZ NSCs | N/A | N/A |
| 293T HEK Cells | ATCC | CRL3216 |
| Experimental Models: Organisms/Strains |  |  |
| Software and Algorithms |  |  |
| Fiji | Schindelin et al., 2012 | https://fiji.sc |
| FastQC | Andrews et al., 2012 | https://www.bioinformatics.babraham.ac.uk/projects/fastqc/ |
