# Supplementary material for: The integrated stress response promotes neural stem cell survival under conditions of mitochondrial dysfunction in neurodegeneration
Source: Aging Cell. 2024 May 16;23(7):e14165. doi: 10.1111/acel.14165 (PMC11258489; doi:10.1111/acel.14165)
Supplement: Supplementary file 3 — Table S2 [file ACEL-23-e14165-s003.docx]

Supplementary Table 2

| ChIP Primers |  |
| --- | --- |
| mSlc3a2-ATF4-pk-F | TGT GAG ACT CCT TGC GTC AG |
| mSlc3a2-ATF4-pk-R | TCC AGC CTC CAC ATT CTC TC |
|  |  |
| mSlc7a11-ATF4bs1-2_F | AAT GTT GGC GCT TTC TCA AG |
| mSlc7a11-ATF4bs1-2_R | CGC CTG TCA CAC CAA CTT AC |
|  |  |
| mChac1_ATF4bs1-2_ChIP_F | AGC CTG ACG CAA TCT GAC TC |
| mChac1_ATF4bs1-2_ChIP_R | CTG ATT GGT TCG GCT CCT C |
|  |  |
|  |  |
| mSlc3a2-H3K4me3-F | GTG TCG TGT CAC CGT TTC TG |
| mSlc3a2-H3K4me3-R | TGC TTC TCC GGT TCT AGC TC |
|  |  |
| mSlc7a11-H3K4me3-F | CTA ACT GAC TGC CCC TGG AG |
| mSlc7a11-H3K4me3-R | GAG GTG TGT TTC AGC CTT CG |
|  |  |
| mChac1-H3K4me3-F | CCC CAA TCA TAG GGA CAG C |
| mChac1-H3K4me3-R | GTC GTT CCA GCT GAG TGG TC |
|  |  |
| qPCR Primers |  |
| Opa1-F | TGG AAT ACA AAG AAA CGT ACC GC |
| Opa1-R | GGG CAG GAT GAT GTG AAC GA |
|  |  |
| Slc3a2-F | TGATGAATGCACCCTTGTACTTG |
| Slc3a2-R | GCTCCCCAGTGAAAGTGGA |
|  |  |
| mSlc7a11_RT_F | GTC TGC CTG TGG AGT ACT GT |
| mSlc7a11_RT_R | ATT ACG AGC AGT TCC ACC CA |
|  |  |
| mCHAC1_RT_F | TGG TGA CCC TCC TTG AAG AC |
| mCHAC1_RT_R | TTG GTC AGG GGT GTC TTG AG |
|  |  |
| Slc1a5 F | GTA AAA TAC CGC AAT CCT GTA TCC |
| Slc1a5 R | CGA TAG CGA AGA CCA CCA GG |
|  |  |
| Xpot f | GCT TCA GGC TCA GAT GCA GA |
| Xpot R | AAA GCA AGG CGA ACA CTT GG |
|  |  |
| Sesn2 Set2 F | GAA GAC GAC CCG TAG GAT GT |
| Sesn2 Set2 R | GCA AGT TCA CAT GAA CCT TCT CT |
|  |  |
| Atf4 f | AAA AGG CAT CCT CCT TGC G |
| Atf4 R | CTT GAT GTC CCC CTT CGA CC |
|  |  |
| Psat1 F | GCT GTC GCC TTA GCA CCA |
| Psat1 R | TGG ATC TCC AAC AAT ACC GAG TG |
|  |  |
| Mthfd2 F | TCC TTG TTG TCT GCG TTG GC |
| Mthfd2 R | CTT CAT TTC GCA CTG CCG CC |
|  |  |
| Aars F | TTG CTA TTC CCT CGG AGC AC |
| Aars R | CTC CTC GGG AAC CTT AGC TC |
|  |  |
| Gars F | GGC AGA GGT CTC TGA GCT G |
| Gars R | GCA CGA TGG TCA TAA GCT GC |
|  |  |
| Cars F | GAG CAG GCT GCC GAC TAC A |
| Cars R | TAT AGC TAC GCG TGC TGA GG |
|  |  |
| Nars F | GAG CCG GCC TGT GTA AAG AT |
| Nars R | GAC CCA GCC AAA CAC CTT CA |
|  |  |
| Iars F | TAT TGC ATC ACC TCC AGA CGC |
| Iars R | TGA ACC ATT CTG TTG CTG GGA |
